# Psychological impact of the quarantine during the COVID-19 pandemic on the general European adult population: a systematic review of the evidence

**DOI:** 10.1017/S2045796022000051

**Published:** 2022-04-27

**Authors:** M. Bonati, R. Campi, G. Segre

**Affiliations:** Laboratory for Mother and Child Health, Department of Public Health, Istituto di Ricerche Farmacologiche Mario Negri IRCCS, 20156 Milan, Italy

**Keywords:** COVID-19 pandemic, quarantine, review, mental health, general population

## Abstract

**Aims:**

Due to the coronavirus disease 2019 (COVID-19) different countries implemented quarantine measures to limit the spread of the virus. Many studies analysed the mental health consequences of restrictive confinement, some of which focused their attention on specific populations. The general public's mental health also requires significant attention, however. This study aimed to evaluate the effects of the COVID-19 quarantine on the general population's mental health in different European countries. Risk and protective factors associated with the psychological symptoms were analysed.

**Methods:**

A systematic search was conducted on four electronic databases (PubMed, PsycINFO, Scopus and Google Scholar). Studies published up until 20th April 2021, and following eligibility criteria were selected for this review. One thousand three hundred thirty-five (1335) studies were screened, 105 of which were included. Via network analysis, the current study investigated the pathways that underlie possible risk factors for mental health outcomes.

**Results:**

Anxiety, depression, distress and post-traumatic symptoms are frequently experienced during the COVID-19 quarantine and are often associated with changes in sleeping and eating habits. Some socio-demographic and COVID-19-related variables were found to be risk factors for an individual's wellbeing. In particular, being female, young, having a low income, being unemployed and having COVID-19-like symptoms or chronic disorders, were found to be the most common risk factors for mental health symptoms.

**Conclusions:**

The COVID-19 pandemic represented an unprecedented threat to mental health globally. In order to prevent psychological morbidity and offer support tailored to short-, medium- and long-term negative outcomes, it is essential to identify the direct and indirect psychosocial effects of the lockdown and quarantine measures, especially in certain vulnerable groups. In addition to measures to reduce the curve of viral transmission, policy makers should urgently take into consideration provisions to alleviate hazards to mental health.

## Introduction

From December 2019 severe acute respiratory syndrome coronavirus 2 (SARS-CoV-2) infection spread rapidly around the world, and in March 2020, the World Health Organization (WHO) declared a global pandemic (World Health Organization, 2020).

The impact of the coronavirus disease was dramatic also because appropriate tools for diagnosis and therapy were not available at the time.

Quarantine has been defined as the separation and restriction of movement of people who have potentially been exposed to a contagious disease to ascertain if they become unwell, so reducing the risk of them infecting others (Centers for Disease Control and Prevention, 2017). Quarantine was used mainly at the local level during historic outbreaks, e.g. during the 2014 Ebola outbreak in African villages.

For coronavirus disease 2019 (COVID-19), quarantine and social distancing measures were effective public health tools in limiting the dissemination and outcomes of the infection (Tognotti, 2013). Although the severity of these restrictions has varied between and within countries, they have had a significant impact on people's daily life, influencing their job, leisure activities, livelihood and social relationships. Each country's general population has experienced the emotional, social and economic impact of this emergency.

Previous studies have shown that widespread outbreaks of infectious diseases, such as SARS, Ebola and H1N1, are associated with psychological distress and mental health symptoms (Bao *et al*., 2020; Maalouf *et al*., 2021; Chaundri *et al*., 2021). The psychiatric implications continued far beyond the outbreak: SARS survivors reported having persistent mental health issues years afterwards (Mak *et al*., 2009).

A review published at the beginning of the COVID-19 pandemic (Brooks *et al*., 2020) showed that quarantines could lead to deleterious psychological effects, including post-traumatic stress symptoms, confusion, anger, infection fears, frustration and boredom.

Several studies have investigated the mental consequences of COVID-19 on target populations such as children, students and healthcare professionals (Husky *et al*., 2020; Xie *et al*., 2020; Segre *et al*., 2021; Stocchetti *et al*., 2021)

While such a focus is understandable, it is also necessary to detect relevant changes in health behaviours that may be occurring at a community level in order to better understand the range of psychosocial consequences of the pandemic's containment measures.

A systematic review and meta-analysis conducted at the beginning of the pandemic (Salari *et al*., 2020) showed that the prevalence of stress, anxiety and depression symptoms among the general population was 30% (95% confidence interval (CI) 24.3–35.4), 32% (95% CI 28–37) and 34% (95% CI 28–41), respectively.

Since lifestyle behaviours can affect mental wellbeing and health behaviours can change during the COVID-19 pandemic (Parletta *et al*., 2016; Arora and Grey, 2020), the potential benefits of mandatory mass quarantine need to be weighed against the possible costs, including psychological ones.

Although the first wave of the pandemic seems far away, two others have followed and others, albeit less intense, may occur. The use of quarantine to deal with epidemics or pandemics, however, may occur again.

Although many studies (Necho *et al*., 2021; Prati *et al*., 2021; Zhang *et al*., 2021) have evaluated the mental health consequences of the current pandemic on the general population, there has been no published systematic review focusing primarily on the broader psychological impact of COVID-19 quarantine on European general population samples.

The main objective of the present study was therefore to investigate the effects of the COVID-19 quarantine during the first wave (the most intense one) on mental health and lifestyle changes of the general population in European countries. Specifically, it aimed to analyse the socio-demographic and COVID-19-related variables in order to identify those individuals at elevated risk for adverse mental health outcomes. Specific focus was placed on pre-quarantine predictors of psychological impact and stressors during quarantine.

## Methods

### Search strategy and selection criteria

For the present review, the Preferred Reporting Items for Systematic Review and Meta-Analysis (PRISMA) guidelines were followed. A computer-based literature search was conducted on the following databases: PubMed, PsycINFO and Scopus, including studies published from the inception of the pandemic (January 2020) until the 20th of April 2021. A manual search on Google Scholar was performed to identify additional relevant studies. The full list of search terms can be found in the Appendix (Table A1). In brief, we used a combination of terms relating to quarantine (e.g. ‘quarantine’, ‘isolation’, ‘confinement’ and ‘lockdown’), psychological outcomes (e.g. ‘psychological’, ‘mental health’, ‘depression’, ‘anxiety’, ‘insomnia’, ‘eating habits’ and ‘lifestyle changes’), survey (e.g. ‘online survey’ and ‘questionnaire’) and COVID-19 (e.g. ‘COVID’, ‘corona-virus’ and ‘pandemic’).

For studies to be included in this review they had to be journal articles, report on primary research, be published in peer-reviewed journals, be written in English, include participants asked to enter into quarantine outside of a hospital environment for at least 24 h and include data on the prevalence of mental health symptoms or psychological wellbeing, or on related factors. In particular, studies with a cross-sectional design and longitudinal studies with data collected only during the quarantine were included. Studies were excluded if they focused on particular subgroups of the population such as healthcare workers, students or people with chronic conditions, or if they did not have full-text availability. The present review followed the PRISMA checklist and reporting guidance (PRISMA-P Group *et al*., 2015).

The titles and abstracts were evaluated by the authors, independently, to decide whether to include or exclude the studies. Disagreements on the eligibility of a study were resolved by discussion until consensus was reached. Moreover, a review of the references of the included studies was performed. Complete references were downloaded and stored using Reference Manager 2011.0.1 software (Thompson Research Soft, Carlsbad, CA, USA).

After the first screening, only studies conducted during the first wave of the pandemic on European countries' general adult population were included. In particular, those living in countries located in the European continent, extending from the island nation of Iceland, in the west, to the Ural Mountains of Russia, in the east, were considered.

### Data analysis

The network analysis approach (Borsboom and Cramer, 2013) was used to investigate the relationship between the 20 variables considered as potential risk factors for mental health outcomes related to COVID-19 quarantine (gender, age, educational level, marital status, parental status, working situation, living conditions during confinement, financial situation, social support, levels of general health, being in a vulnerable group, pre-existing mental health disorder, working situation, changes in diet and nutrition, changes in sleep, physical activity during quarantine, living in specific areas during the pandemic, symptoms of COVID-19/Physical symptoms, contact with COVID-19 cases, coping strategies/strategies to deal with stress). The Fruchterman–Reingold algorithm was used (Fruchterman and Reingold, 1991), in which a force-directed layout dissembles the graph as a system of a large quantity of nodes or vertices. Psychological distress is seen as a network of specific risk factors (termed nodes) that dynamically interact with, and impact, one another. The nodes represent the 20 variables considered and the edges represent the connections between the nodes. Nodes act as mass particles and edges behave as springs between the nodes. The degree of a node is its number of connections (how many neighbours the variable has with other variables). The figure generated shows the most consistent associations, where thicker edges show stronger relationships and thinner edges weaker relationships.

For each node, we calculated:

- *betweenness centrality*, which measures all the shortest paths between every pair of nodes of the network and then counts how many times a node is on a shortest path between two others,
- *closeness centrality*, which calculates the shortest paths between all nodes, then assigns each node a score based on its sum of shortest paths,
- *eigen centrality*, which measures a node's influence based on the number of links it has to other nodes in the network and can identify nodes with an influence over the whole network, not just those directly connected to it.

A community detection analysis was carried out using the Louvain method (Blondel *et al*., 2008) to extract communities and calculate modularity. It is one of the most frequently used methods for clustering on large networks, it is very efficient and allows one to define communities in a hierarchical way to group together certain nodes, diminish the dimensionality of a dataset and facilitate interpretability. Network analysis was performed using Gephi version 0.9.2 (Bastian *et al*., 2009).

Methodological quality/bias risk were recorded using the Joanna Briggs Institute critical appraisal checklist for cross-sectional and cohort studies (see Appendix Tables A2 and A3).

## Results

**Fig. 1.**
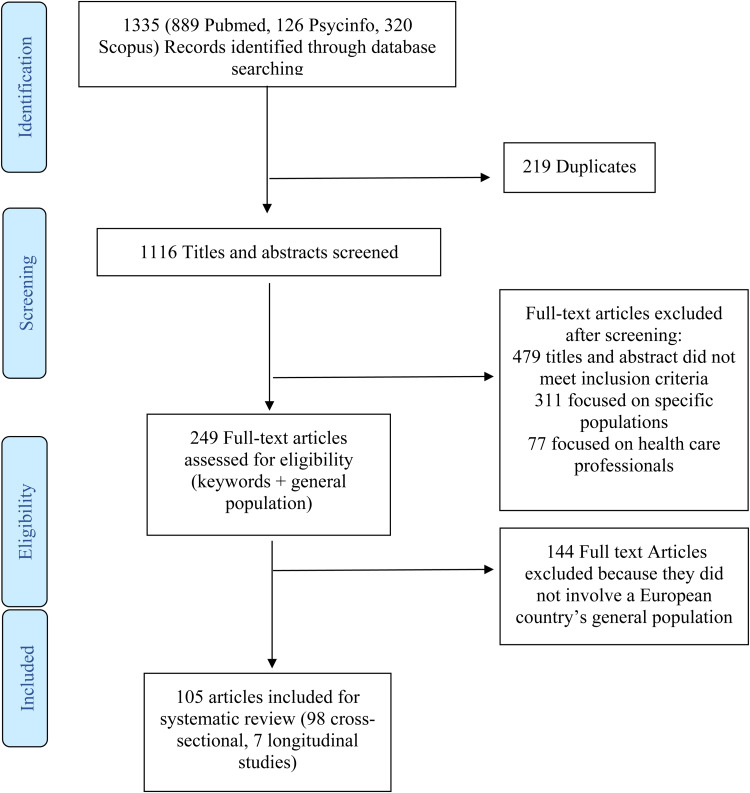
Study selection.

Figure 1 presents the procedural steps adopted and the record count, duplicates and final studies obtained after screening. The initial search yielded 1335 studies, of which 105 included relevant data and were included in this review.

An overview with the characteristics of the studies is presented in online Supplementary Tables 1 and 2.

All eligible studies were included in the review, regardless of their quality assessment results. Of the 98 cross-sectional studies, 45 studies (46%) were of very good quality (maximum score on the JBI) and 8 (9%) were of poor quality (JBI score <5 points). All cohort studies were of good quality. Selected studies were conducted mainly in 17 different countries (Italy: *n* = 39, Spain: *n* = 25, UK: *n* = 9 and Greece: *n* = 4).

### The psychological impact of quarantine

Seventy-nine studies reported anxiety symptoms in the general population, with a prevalence ranging from 5.5 to 70.4%. The highest levels of anxiety were found in an Italian study (Di Renzo *et al*., 2020); these involved 70.4% of the enrolled population, 57.8% of whom with physical manifestations of anxiety (tachycardia, headache, sweating). On the contrary, three studies (Bonati *et al*., 2021; Budimir *et al*., 2021; Silva Moreira *et al*., 2021) found low percentages of anxiety symptoms (<10% of the sample).

A comparative investigation between Spanish and Greek participants (Papandreou *et al*., 2020) observed a similar prevalence of moderate and severe anxiety symptoms, with 12.3% in Spain and 13.2% in Greece. Similar rates were found also in a German study (Munk *et al*., 2020), in which 12% of the sample met the criteria for the general anxiety disorder (GAD) during the lockdown, compared with 2% before the pandemic.

Depressive symptomatology and mood variables were assessed in 74 studies and their clinical prevalence ranged from 3.2 to 82.6%. Sixteen studies classified the frequency and severity of symptoms in three categories: mild, moderate and severe. The lowest percentages of severe depressive symptoms were found in 3.2% of the Austrian sample (Budimir *et al*., 2021) and 9.3% of the Greek sample (Fountoulakis *et al*., 2021). Close rates were reported in the Portuguese population (Paulino *et al*., 2021) in which only 11.7% of the participants presented moderate to severe depressive symptoms on the ‘Depression, Anxiety and Stress Scale’ (DASS). On the contrary, the findings of a Polish study (Bodecka *et al*., 2021) showed that the majority of participants displayed at least mild depressive symptoms (82.6%). Nearly two-thirds of the Italian respondents (61.3%) experienced depressed mood (Di Renzo *et al*., 2020).

Psychological distress has been assessed with different tools: the majority of the included studies used the DASS stress scale. Four Italian studies (Costantini and Mazzotti, 2020; Landi *et al*., 2020; Pakenham *et al*., 2020; Bonati *et al*., 2021) used the ‘COVID-19 Peritraumatic Distress Index (CPDI)’ with positive responses ranging from 15 to 40%. Nearly one-third of people experienced symptoms of mild to moderate and severe peritraumatic distress in two studies (Costantini and Mazzotti, 2020; Pakenham *et al*., 2020), while lower rates (15.5% of the sample) were reported in another study (CPDI mean 17.95, s.d. 11.50) (Landi *et al*., 2020).

Eighteen studies focused their attention on post-traumatic stress disorder (PTSD) symptoms. In total, 54.4% of the Italian participants met criteria for a clinical level of stress related problems and 30% of the sample had probable diagnosis of PTSD (Panno *et al*., 2020). Lower scores of PTSD (5.1%) were reported in a study (Favieri *et al*., 2021) that specifically used the Post-Traumatic Stress Disorder Related to COVID-19 (COVID-19-PTSD). High levels of avoidance symptoms at the Impact of Events Scale-Revised (IES-R) were found in two studies (Fiorillo *et al*., 2020; Jiménez *et al*., 2020).

Seventeen studies focused specifically on resilience and/or coping strategies, i.e. the individual's ability to cope with stress and adapt to changes. Resilience has been associated with a lower risk for any mental health symptoms; the same results were obtained regarding coping (Munk *et al*., 2020). A higher score on the positive coping strategy dimension was associated with a lower prevalence of depressive symptoms, while more supportive/distractive strategies were associated with an increased prevalence (Skapinakis *et al*., 2020).

### Pre-quarantine predictors of psychological impact

Several predictive factors were identified from the included studies.

Female gender is the most common risk factor associated with psychological symptoms during the COVID emergency. The risk of developing anxiety, depression, distress symptoms or PTSD was double in female compared to male participants (Casagrande *et al*., 2020; Fiorillo *et al*., 2020; Gualano *et al*., 2020; Landi *et al*., 2020; Mariani *et al*., 2020; Mazza *et al*., 2020; Pieh *et al*., 2020; Rodríguez-Rey *et al*., 2020; Suso-Ribera and Martín-Brufau, 2020; Bonati *et al*., 2021; Rettie and Daniels, 2021). On the contrary, a Spanish study reported similar levels of anxiety, stress and depression (Ozamiz-Etxebarria *et al*., 2020). Women reported more frequent and severe sleeping problems (such as insomnia) than men (Bacaro *et al*., 2020; Casagrande *et al*., 2020; Margetić *et al*., 2021); they exhibited more PTSD or secondary traumatic stress and posttraumatic growth, were less resilient and used all kinds of coping strategies more often (Kalaitzaki, 2021).

An age-related variation was analysed in different studies: the psychological impact of COVID-19 confinement seems to ameliorate as people get older. The youngest participants (<35 years old) showed higher levels of depression, anxiety, stress, insomnia and PTSD symptoms compared to the other age groups (Antunes *et al*., 2020; Bacaro *et al*., 2020; Bonsaksen *et al*., 2020; Rodríguez-Rey *et al*., 2020; Paulino *et al*., 2021; Rettie and Daniels, 2021; Rossi *et al*., 2021). The greater vulnerability to distress in young adulthood could be due to the precariousness of the working activities, with consequent interruption of income, and/or the interruption of the initial phase of development of one's professional activity, and/or the presence of children, with resulting age-related concerns or the forced cohabitation in a phase in which young adults would normally leave the family of origin. Age remained positively associated with wellbeing and negatively associated with depression (Dawson and Golijani-Moghaddam, 2020): marked differences in prevalence of depression were found between 18 and 24 year (63.3%) olds and people over 65 years of age (11.5%) (Pieh *et al*., 2021). A similar pattern, even if slighter, was reported for sleep problems (Gualano *et al*., 2020; Beck *et al*., 2021). Results of a Spanish (Vicario-Merino, 2020) and a UK study (Neill *et al*., 2021) stated that symptoms of stress and depression tended to increase with an increase in age range.

Being more educated predicted greater wellbeing: lower educational status was significantly associated with higher depression, anxiety and PTSD symptoms (Benke *et al*., 2020; Di Crosta *et al*., 2020; Haesebaert *et al*., 2020; Skapinakis *et al*., 2020; Suso-Ribera and Martín-Brufau, 2020; Gutiérrez-Hernández *et al*., 2021; Silva Moreira *et al*., 2021). The trend of the association with education level, however, is likely also related also to the cultural context, as found in Italian results (Bonati *et al*., 2021) *v.* Portuguese ones (Paulino *et al*., 2021).

Moreover, having a partner also predicted greater wellbeing (Haesebaert *et al*., 2020): married participants and those cohabiting with their partner showed significantly lower psychological impact and felt less lonely than single participants (Balsamo and Carlucci, 2020; Cerbara *et al*., 2020; Saita *et al*., 2021). Although, an Italian study (Velotti *et al*., 2021) reported that having a partner was associated with overeating and social network use during the quarantine, sharing everyday life with someone during quarantine was a protective factor (Dawson and Golijani-Moghaddam, 2020; Gualano *et al*., 2020).

Additionally, living with children in the household was revealed as a protective factor against psychological distress, anxiety and depressive symptoms in five different studies (Gómez-Salgado *et al*., 2020; Mazza *et al*., 2020; Rodríguez-Rey *et al*., 2020; Ellen and De Vriendt Patricia, 2021; Saita *et al*., 2021). In particular, a low rate of psychological distress was observed among people living with older children or adolescents (Gómez-Salgado *et al*., 2020; Rodríguez-Rey *et al*., 2020), but those living with children under ten had poorer wellbeing (Haesebaert *et al*., 2020).

The impact of confinement was more damaging for people living in very poor cohabitation conditions. In particular, participants living in houses of more than 120 square meters showed lower psychological impact, stress, anxiety and depression symptoms than those living in less than 30 square meters (odds ratio (OR) 1.98, 95% CI 1.19–3.30) (Ramiz *et al*., 2021). Moreover, people with access to an outdoor space (e.g. garden, balcony) had higher wellbeing scores (OR 1.38, 95% CI 1.00–1.89) (Ramiz *et al*., 2021) and better mental health (Haesebaert *et al*., 2020; Ellen and De Vriendt Patricia, 2021). Both the number of cohabitants and the quality of the relationships must be taken into account, however, levels of psychological distress were higher and sleep quality was lower in people living alone (Pérez *et al*., 2021).

Being affected by a pre-existing mental disorder or having a pre-existing physical disease were found to be factors associated with worse levels of depressive and anxiety symptoms (Fiorillo *et al*., 2020; Mazza *et al*., 2020; Pérez *et al*., 2021; Rettie and Daniels, 2021). In particular, people in ‘vulnerable’ groups were significantly more anxious, and more anxious concerning their health, compared to individuals in nonvulnerable groups (Rettie and Daniels, 2021).

### Stressors during quarantine

Unemployed participants, who were more vulnerable to the possible economic crisis in the pandemic's aftermath, presented higher rates of depression, anxiety and stress symptoms compared to employed participants (Benke *et al*., 2020; Pieh *et al*., 2020; Bonati *et al*., 2021; Paulino *et al*., 2021). Unemployed participants were also at higher risk of developing sleep disorders (68%), often associated with some impairment of their daytime daily activities (OR 1.34; 95% 95% CI 1.02–1.70) (Casagrande *et al*., 2020; Beck *et al*., 2021). Working outside the home was associated with higher levels of psychological distress: people working in-presence showed significantly higher psychological impact compared to those working remotely (Di Giuseppe *et al*., 2020; Gómez-Salgado *et al*., 2020; Mazza *et al*., 2020; Paulino *et al*., 2021), although the type of job and professional role may affect the relationship (Fiorenzato *et al*., 2021). Economic stability, and socioeconomic status in general, are related to depression, anxiety and PTSD symptoms (Di Crosta *et al*., 2020; Prati, 2021): participants with high monthly family income showed lower psychological impact than those whose family income was lower (Nese *et al*., 2022; Pieh *et al*., 2020, 2021; Skapinakis *et al*., 2020; Pérez-Rodrigo *et al*., 2021).

Health became one of the primary concerns during the COVID-19 confinement. Symptomatic individuals expressed higher psychological impact and increased levels of depression, anxiety, stress symptoms and sleep disorders; these symptoms could be interpreted as potential symptoms of COVID-19 (Beck *et al*., 2021; Paulino *et al*., 2021; Vujčić *et al*., 2021). Patients with polymerase chain reaction-confirmed COVID-19 reported greater sleep problems (52% severe) and worse levels of depressive and anxiety symptoms (Fiorillo *et al*., 2020). Having had a contact with a positive case in the previous 14 days showed a statistically significant relationship with the presence of psychological distress (Gómez-Salgado *et al*., 2020).

Home confinement affected habits and lifestyle (in terms of sleep disorders, food consumption and physical activity), inducing common mental health problems (Balanzá-Martínez *et al*., 2021).

In total, 74% of the participants of a French study reported trouble sleeping, of whom females and the young had greater frequency and severity (Beck *et al*., 2021). Reduced sleep, poor sleep quality and changes in usual sleep patterns were associated with more negative mood and anxiety symptoms (Bacaro *et al*., 2020; Suso-Ribera and Martín-Brufau, 2020). Adhering to a routine, maintaining the same weight and moderate physical exercise were associated with fewer negative effects, indicating that they are important protective factors (Gismero-González *et al*., 2020). Age was inversely related to dietary control, and being female was associated with being more anxious and disposed to eating comfort food than males (Di Renzo *et al*., 2020).

Increased emotional eating was predicted by higher depressive and anxiety symptoms, quality of personal relationships and quality of life, while an increase in bingeing was predicted by higher stress (Cecchetto *et al*., 2021).

The respondents who maintained the same physical activity habits had higher levels of positive emotions (energy), lower levels of negative emotions (fear and anxiety) and lower levels of experienced symptoms (headache and fatigue) (Di Corrado *et al*., 2020). Increased duration and greater intensity of physical activity were both associated with further reduction in the prevalence of depression (Jacob *et al*., 2020; Pieh *et al*., 2020), in particular in females, suggesting that variations in physical activity habits may have more influence in women's psychological status than in men's (Maugeri *et al*., 2020).

## Network analysis

**Fig. 2.**
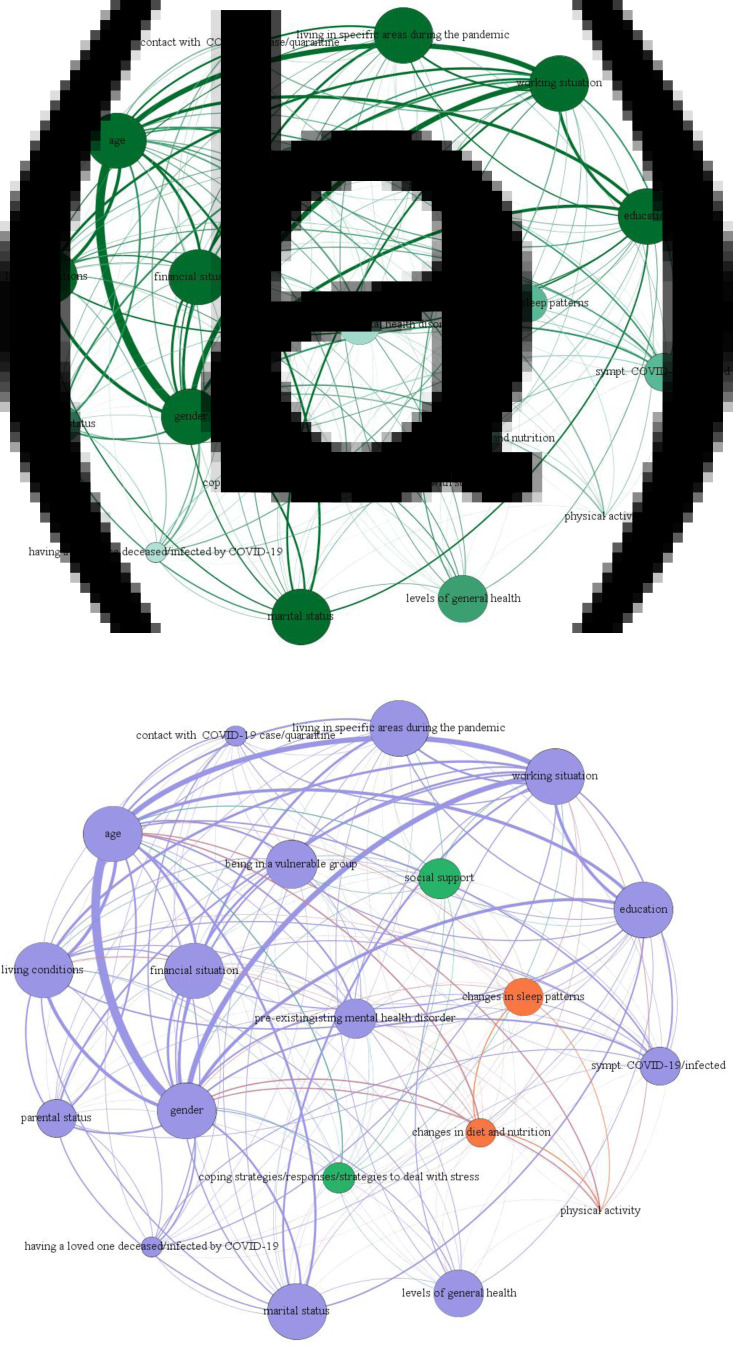
Network analysis.

The connections between the 20 prevalent variables analysed in the retrieved studies and the structure of the network are shown in Fig. 2a, where the diameter of the node refers to the degree centrality and the hue of the node refers to betweenness centrality (darker = higher value). The network had 330 non-zero edges out of 380 possible edges. The weights of the connections are presented in online Supplementary Table 3.

In agreement with the narrative analysis reported above, the strongest connection emerged between gender and age, meaning that these were found to be the most common risk factors for psychological distress during quarantine. A cluster was found between age, gender, living in specific areas and working situation during the pandemic. Other noteworthy associations were reported between gender and working situation, age and living area, and working situation and living area, during the quarantine.

Figure 2b shows the results of the community detection analysis, where the colour of the node refers to the partition of the network. All nodes related to socio-demographic characteristics (gender, age, working situation, living condition, financial situation and marital status) and variables related to health (symptoms of COVID-19/physical symptoms/being infected by COVID-19, pre-existing mental health disorder) formed one large module (node in violet). Two nodes were found outside this large module: the first comprised of changes in diet and nutrition, changes in sleep patterns and physical activity (node in orange); the second one (node in green) was related to coping strategies/responses/strategies to deal with stress and social support.

**Fig. 3.**
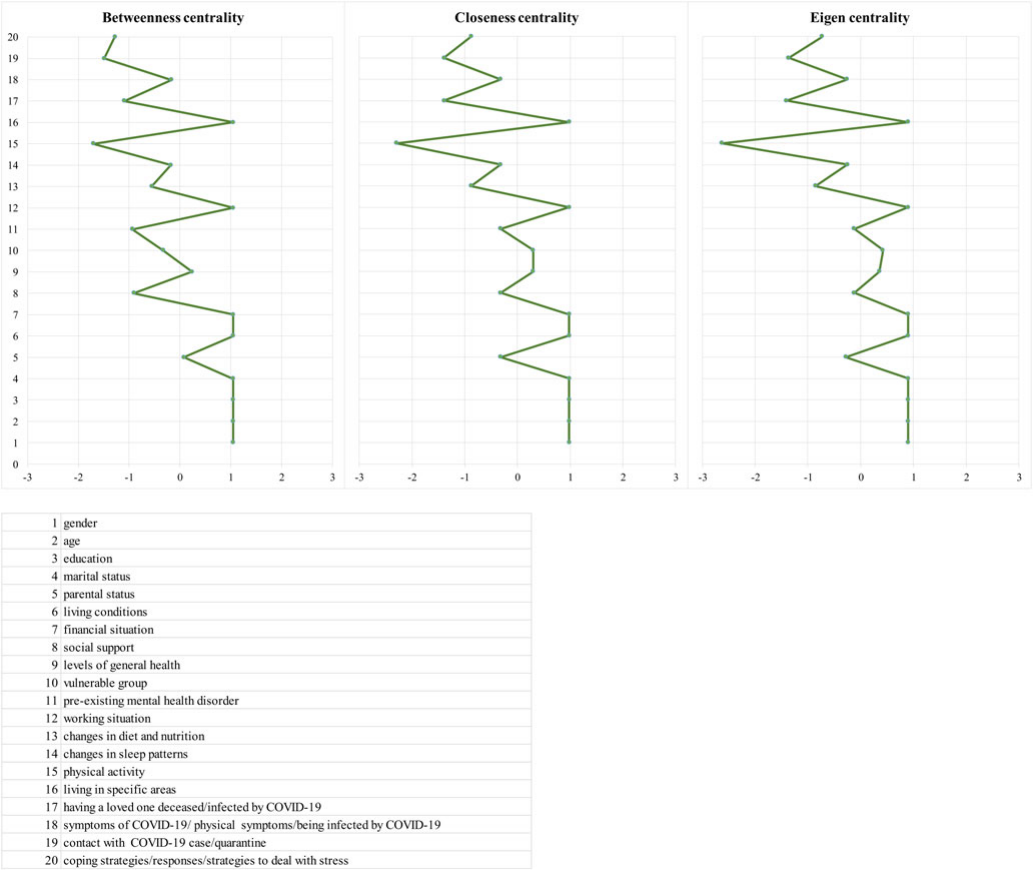
Centrality measures (centrality metrics are shown as standardised *z*-scores).

Figure 3 shows the resulting plot for centrality metrics, which highlights the differences in connectivity of the network.

The three indices are significantly intercorrelated with each other: the correlation between eigen and closeness is 0.99 (*p* < 0.01), the correlation between eigen and betweenness is 0.91 (*p* < 0.01) and the correlation between closeness and betweenness is 0.95 (*p* < 0.01).

Gender, age, education, marital status, living conditions, financial situation, working situation and living in specific areas have the highest betweenness, closeness and eigen strengths, being the most central nodes, suggesting that they have the most connections in the network.

All the centrality measures indicate that the most central isolation variables in the network are physical activity, contact with COVID-19 case/quarantine and coping strategies/responses/strategies to deal with stress. Parental status, levels of general health, changes in sleep patterns and symptoms of COVID-19/physical symptoms/being infected by COVID-19 are the most central variables in the distribution of the three *z*-scored centrality metrics.

## Discussion

This systematic review has analysed data from different studies that investigated the psychological impact of the quarantine on the general European population during the first wave of the SARS-CoV-2 pandemic.

Similar to those of other reviews (Luo *et al*., 2020; Salari *et al*., 2020), the findings of the present study highlight the fact that anxiety, depression, distress and post-traumatic symptoms were frequently experienced during the COVID-19 quarantine and were often associated with changes in sleeping and eating habits. In particular, the overall effect of the pandemic has been linked with worsening psychiatric symptoms. The long-term effect of direct COVID-19 infection has, however, been associated with no, or mild, symptoms (Bourmistrova *et al*., 2022)

These data should be interpreted with caution since different studies reported a considerable heterogeneity of mental health problems: the impact of the COVID-19 pandemic may have been different across different social groups and across different contexts and countries.

An increase in mental health problems was seen from pre-pandemic assessments through the first phase of lockdown; during lockdown, no uniform trend could be identified and after lockdown, mental health problems decreased slightly (Richter *et al*., 2021).

Similarly, another recent review (Robinson *et al*., 2022) observed an increase in mental health symptoms among most population sub-groups and symptom types soon after the outbreak of the COVID-19 pandemic, which then decreased and were comparable to pre-pandemic levels by mid-2020.

On the contrary, a relatively small effect of lockdowns on mental health was reported (Prati and Mancini, 2021) providing evidence of people's robust capacity for resilience.

Several issues should be kept in mind when interpreting the findings of the current study.

Only studies written in English have been considered in the present review, and this may have led to some bias, although a study conducted in 2012 (Morrison *et al*., 2012) showed that little evidence of bias was introduced from the exclusion of non-English studies.

Some of the selected studies were conducted during the initial stages of the COVID-19 outbreak; it is therefore possible that they underestimated the actual occurrence of traumatic stress in the population, since delayed onset of symptoms, especially PTSD ones, is conceivable. Moreover, data collection time for cross-sectional studies (online Supplementary material, Fig. 4) differed also because decisions concerning time and type of quarantine differed between European countries.

**Fig. 4.**
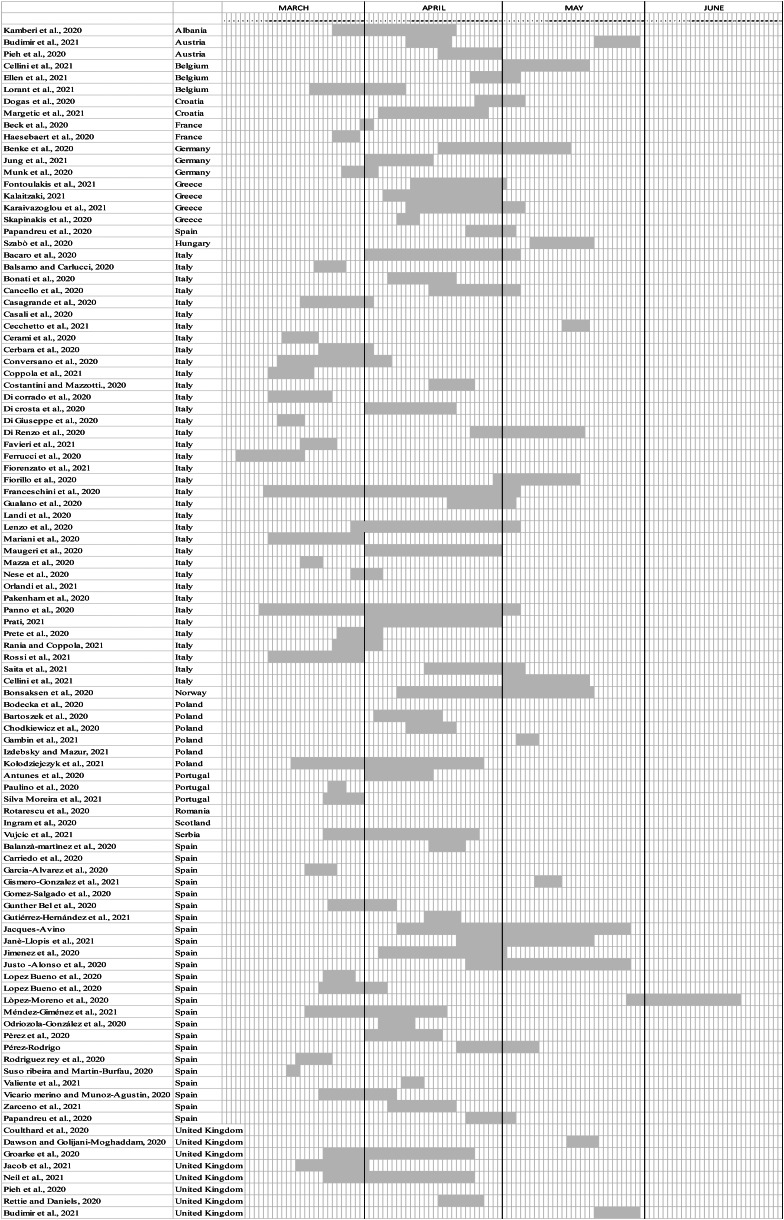
Timing of data collection for each European country.

The majority (98 out of 105) of the selected studies had a cross-sectional observational design, which does not allow one to establish cause and effect relationships and temporal association between variables, so these should be interpreted with caution. Time-limited, cross-sectional survey data shed little light on the enduring effects of quarantine, on how adaptations to quarantine changed or evolved over time, and on what happened during re-opening, when home-confinement restrictions began to ease. Only a few studies (Ozamiz-Etxebarria *et al*., 2020; Salfi *et al*., 2020; Ausín *et al*., 2021; Cheval *et al*., 2021; O'Connor *et al*., 2021; Velotti *et al*., 2021; Zavlis *et al*., 2021) analysed data at different time-points during the restrictive measures, in order to investigate the psychological impact caused by the pandemic longitudinally.

Considering that previous research on the long-term effects of pandemics and quarantining (Brooks *et al*., 2020) has shown that not only acute mental health effects occur, but that psychological distress may persist long after the crisis, it is essential to prioritise studies with longitudinal designs.

It is thus imperative to prospectively document the synergistic effects of multiple co-occurring risk factors, such as economic precarity, unemployment, isolation, uncertainty, loss, and fear, which may increase the likelihood of mental health difficulties. It is also important to highlight the fact that the effects of stress exposure may not manifest themselves immediately, but, in some individuals, may unfold over time (Wade *et al*., 2020; Veldhius *et al*., 2021).

Moreover, the processes that cultivate resilience change dynamically over time, and this supports the fact that the pandemic requires longitudinal analyses in order to monitor individual adaptation to uncertain conditions.

Only four studies (Szabó *et al*., 2020; Castellini *et al*., 2021; Lorant *et al*., 2021; Ramiz *et al*., 2021) compared data collected during the pandemic's quarantine with the level of psychological status found in the general population under normal conditions.

Concerning the assessment tools, the majority of the studies used validated and reliable assessment instruments in order to investigate several domains of mental health and psychological wellbeing. Different assessment scales were used for population screening and different cut offs were employed by studies that used the same tests. The self-report questionnaires used in the majority of the studies were ‘the Patient Health Questionnaire’ (PHQ), used in 29 studies, and the GAD, used in 27 studies. Seven studies created *ad hoc* questionnaires (Cancello *et al*., 2020; Cerbara *et al*., 2020; Di Corrado *et al*., 2020; Đogaš *et al*., 2020; Ferrucci *et al*., 2020; Nese *et al*., 2022; Izdebski and Mazur, 2021). It must be noted that data collected relied on self-report measures related to psychological symptoms, and thus cannot be considered sufficient to formulate diagnoses of specific disorders.

The degree to which self-reported prevalence rates effectively represent common distress is still unknown, as well as to what extent this distress will result in increased rates of mental disorders and need for subsequent health treatment (Richter *et al*., 2021).

Although the symptomatology was assessed with widely used screening tools, scores should not be confused with a diagnosis, which can be assessed only by mental health professionals with additional assessment methods such as structured clinical interviews. It is important to note that the increase in psychological distress during quarantine is related to subjective perception and that there is a lack of pre-post pandemic analyses.

Another relevant aspect that should be considered is the possibility of selection bias related to the use of online surveys. The use of online surveys, and the snowball method for increasing participation, limit the generalisability of the results, although surveys currently represent the best methodological choice for data collection in a short time and in a pandemic situation. The convenient non-probabilistic nature of the chosen sample may not represent the countries' general populations: use of an online tool limits the participation of persons who do not use this type of technology, penalising, for example, elderly people or those living in socially disadvantaged contexts. Moreover, it was not possible to assess the participation rate since the number of subjects who received the link to the surveys was unknown.

A possible gender-related effect, which may not have been identified due to the small number of men who responded, should also be taken into consideration. More women than men participated in the studies, coherently with previous research, reaffirming that it is more difficult to recruit male participants (Korkeila *et al*., 2001; Dunn *et al*., 2004). Furthermore, variable distribution might differ between a sample and the population of reference for residence, age, sex, education and other characteristics, and this requires that study findings be generalised with caution.

Lastly, more than half of the studies enrolled Italian and Spanish populations: 40 studies collected data from Italy and 26 from Spain. This represents an unbalanced interest compared with other European countries, although the severity of COVID-19 in the two Mediterranean countries from the beginning of the pandemic can, in part, justify such a huge production. All the questionnaires were launched nation-wide but, at the time of data collection, the COVID-19 outbreak was more severe in some countries and in specific regions. This may have motivated more people living in those areas to fill in the questionnaires compared to residents from other regions. Moreover, COVID-19 has had different mortality rates worldwide, and the severity and frequency of mental health outcomes could be related to the intensity of the viral spread.

### What can be done to mitigate the consequences of quarantine?

The current COVID-19 health emergency has completely changed the daily life of the population. Both the confinement scenario and the spread of the virus, as well as associated consequences, could alter people's cognitive and emotional state through perceived threat from the virus and through development of negative affective balance and feelings. Several individual, economic and psychological factors have also been found to play a role in the development of higher levels of symptomatology.

The pandemic has highlighted the need to pay greater attention to gender and to the private sphere to prevent, and alleviate, the psychological consequences of pandemic on more vulnerable groups.

Despite the limitations of the retrieved studies, justified in part by the need to rapidly to assess the situation as a whole, our results highlight the importance of identifying which groups may face more difficulties in adopting healthy behaviours (e.g. physical activity, healthy food choices and sleep routines) and maintaining physical and psychological wellbeing. By identifying vulnerable groups, intervention strategies may be more targeted, and the effectiveness of health strategies may be improved.

Maintaining regular habits during restrictive measures could be considered a protective factor for mental health outcomes. Encouraging healthy food choices, regular mealtimes and the carrying out of physical activity at home could therefore be a useful strategy to make the population aware of the need to remain healthy. The promotion of correct lifestyles is important for the protection of health, but it becomes even more important during periods of forced home confinement in reducing long-term negative effects of quarantine. Suggestions on how to maintain a correct lifestyle could be provided through video or app-based supports, but also through non-digital channels (such as TV, newspapers, journals, posters or leaflets) in order to reach less technology-oriented people.

Given that the most effective healthcare measure for reducing the incidence of the coronavirus pandemic was quarantine, and the fact that globalisation and travel increase the likelihood that a similar situation may occur in the future, knowledge of the emotional and cognitive effects of quarantine on the population could lead to the implementation of more effective measures aimed at facilitating coping strategies.

It is essential to implement psychoeducational programmes to manage the emotional and affective alterations caused by restrictive measures, especially if they are taken on a mass level and are repeated in time.

## Conclusions

The implementation of forced restrictive measures to prevent the spread of the COVID-19 infection, in particular the more limiting ones such as quarantine, has influenced individual mental health. Depression, anxiety, psychological distress and post-traumatic symptoms have been the predominant, new-onset psychological health problems in European general populations during the pandemic. Several risk factors have been identified, such as being female, young, having a low income, being unemployed and having COVID-19-like symptoms.

Overall, despite the limitation of the studies, due also to the emergency pandemic situation, the results of this review suggest that there is an immediate psychological impact of the quarantine. Concerning the long-lasting effects, this impact may depend on each country's strategies and duration of restrictive measures taken. To mitigate the significant negative effects on emotional wellbeing, the adoption of appropriate strategies by health services is fundamental, as is preparing the general population for possible future waves of the pandemic. When applying quarantine measures, policy makers should attempt to find the right balance between reducing the risk of infection and minimising the risk of negative mental consequences, while also empowering wellbeing, especially in vulnerable groups.

Future research, based on longitudinal analyses, should attempt to monitor the increase in mental health symptoms over time, in particular their course after the end of the restrictive measures. It would also be important to investigate the social context-related factors that are likely to influence their relationship with quarantine.

Moreover, in addition to providing a focus on the most vulnerable populations, research should investigate between-country variations that result from the confluence of specific environmental stressors and time and type of quarantine in that given area.

## Supplementary material

For supplementary material accompanying this paper visit https://doi.org/10.1017/S2045796022000051. click here to view supplementary material

## Availability of data and materials

The data are available from the authors upon request.

## Appendix

**Table A1.** Keywords used for searching databases

Search query	Keywords (searched within titles, abstracts and general keywords)
1	‘quarantine’ OR ‘isolation’ OR ‘isolate’ OR ‘confinement’ OR ‘Lockdown’ OR ‘home quarantine’ OR ‘ quarantined’
2	‘COVID’ OR ‘COVID-19’ OR ‘nCoV’ OR ‘corona-virus’ OR ‘outbreak’ OR ‘epidemic’ OR ‘pandemic’ OR ‘coronavirus’ OR ‘Sars-cov-2’
3	‘mental health’ OR ‘mental disorders’ OR ‘mental illness’ OR ‘psychiatric’ OR ‘psychological’ OR ‘psychosocial’ OR ‘mental wellbeing’ OR ‘depression’ OR ‘depressive’ OR ‘sleep disorder’ OR ‘insomnia’ OR ‘anxiety’ OR ‘PTSD’ OR ‘distress’ OR ‘affective’ OR ‘fear’ OR ‘phobia’
4	‘Survey’ OR ‘questionnaire’ OR ‘online survey’ OR ‘self-report questionnaire’
Final search query	1 AND 2 AND 3 AND 4 AND 4

**Table A2.** Critical appraisal of cross-sectional studies

Study	Johanna Briggs Institute Score (Moola *et al*., 2020)	Were the criteria for inclusion in the sample clearly defined?	Were the study subjects and the setting described in detail?	Exposure measured in a valid and reliable way?	Objective, standard criteria used for measurement of the condition?	Confounding factors identified?	Strategies to deal with confounding factors stated?	Outcomes measured in a valid and reliable way?	Appropriate statistical analysis used?
**Antunes *et al*. (2020)**	6	Y	Y	Y	Y	N	N	Y	Y
**Bacaro *et al*. (2020)**	8	Y	Y	Y	Y	Y	Y	Y	Y
**Balanzà-Martìnez *et al*. (2021)**	8	Y	Y	Y	Y	Y	Y	Y	Y
**Balsamo and Carlucci (2020)**	7	N	Y	Y	Y	Y	Y	Y	Y
**Bartoszek *et al*. (2020)**	8	Y	Y	Y	Y	N	N	Y	Y
**Beck *et al*. (2021)**	3	N	Y	N	N	N	N	Y	Y
**Benke *et al*. (2020)**	5	N	Y	N	N	Y	Y	Y	Y
**Bodecka *et al*. (2021)**	4	N	Y	Y	Y	N	N	Y	N
**Bonati *et al*. (2021)**	7	N	Y	Y	Y	Y	Y	Y	Y
**Bonsaksen *et al*. (2020)**	8	Y	Y	Y	Y	Y	Y	Y	Y
**Budimir *et al*. (2021)**	6	N	Y	Y	Y	N	Y	Y	Y
**Cancello *et al*. (2020)**	5	N	Y	N	N	Y	Y	Y	Y
**Carriedo *et al*. (2020)**	7	N	Y	Y	Y	Y	Y	Y	Y
**Casagrande *et al*. (2020)**	8	Y	Y	Y	Y	Y	Y	Y	Y
**Casali *et al*. (2021)**	8	Y	Y	Y	Y	Y	Y	Y	Y
**Castellini *et al*. (2021)**	8	Y	Y	Y	Y	Y	Y	Y	Y
**Cecchetto *et al*. (2021)**	7	N	Y	Y	Y	Y	Y	Y	Y
**Cellini *et al*. (2021)**	7	N	Y	Y	Y	Y	Y	Y	Y
**Cerami *et al*. (2020)**	8	Y	Y	Y	Y	Y	Y	Y	Y
**Cerbara *et al*. (2020)**	5	N	Y	N	N	Y	Y	Y	Y
**Chodkiewicz *et al*. (2020)**	4	N	Y	Y	Y	N	N	Y	N
**Conversano *et al*. (2020)**	7	N	Y	Y	Y	Y	Y	Y	Y
**Coppola *et al*. (2021)**	8	Y	Y	Y	Y	Y	Y	Y	Y
**Costantini and Mazzotti (2020)**	7	N	Y	Y	Y	Y	Y	Y	Y
**Coulthard *et al*. (2020)**	8	Y	Y	Y	Y	Y	Y	Y	Y
**Dawson and Golijani-Moghaddam (2020)**	8	Y	Y	Y	Y	Y	Y	Y	Y
**Di Corrado *et al*. (2020)**	2	Y	Y	N	N	N	N	N	N
**Di Crosta *et al*. (2020)**	7	N	Y	Y	Y	Y	Y	Y	Y
**Di Giuseppe *et al*. (2020)**	8	Y	Y	Y	Y	Y	Y	Y	Y
**Di Renzo *et al*. (2020)**	8	Y	Y	Y	Y	Y	Y	Y	Y
**Đogaš *et al*. (2020)**	6	Y	Y	Y	Y	N	N	Y	Y
**Ellen *et al*. (2021)**	8	Y	Y	Y	Y	Y	Y	Y	Y
**Favieri *et al*., 2021**	6	Y	Y	Y	Y	N	N	Y	Y
**Ferrucci *et al*. (2020)**	5	N	Y	N	N	Y	Y	Y	Y
**Fiorenzato *et al*. (2021)**	8	Y	Y	Y	Y	Y	Y	Y	Y
**Fiorillo *et al*. (2020)**	7	N	Y	Y	Y	Y	Y	Y	Y
**Fountoulakis *et al*. (2021)**	7	N	Y	Y	Y	Y	Y	Y	Y
**Franceschini *et al*. (2020)**	8	Y	Y	Y	Y	Y	Y	Y	Y
**Gambin *et al*. (2021)**	7	N	Y	Y	Y	Y	Y	Y	Y
**García-Álvarez *et al*. (2020)**		Y	Y	Y	Y	Y	Y	Y	Y
**Gismero-González *et al*. (2021)**	6	Y	Y	Y	Y	Y	N	N	Y
**Gomez-Salgado *et al*. (2020)**	8	Y	Y	Y	Y	Y	Y	Y	Y
**Groarke *et al*. (2020)**	8	Y	Y	Y	Y	Y	Y	Y	Y
**Gualano *et al*. (2020)**	7	N	Y	Y	Y	Y	Y	Y	Y
**Gutiérrez-Hernández *et al*. (2021)**	8	Y	Y	Y	Y	Y	Y	Y	Y
**Gunther Bel *et al*. (2020)**	8	Y	Y	Y	Y	Y	Y	Y	Y
**Haesebaert *et al*. (2020)**	8	Y	Y	Y	Y	Y	Y	Y	Y
**Izdebski and Mazur (2021)**	5	N	Y	N	N	Y	Y	Y	Y
**Jacob *et al*. (2020)**	8	Y	Y	Y	Y	Y	Y	Y	Y
**Jacques-Avino *et al*. (2020)**	8	Y	Y	Y	Y	Y	Y	Y	Y
**Janè-Llopis *et al*. (2021)**	8	Y	Y	Y	Y	Y	Y	Y	Y
**Jiménez *et al*. (2020)**	5	N	Y	Y	Y	N	N	Y	Y
**Jung *et al*. (2020)**	4	N	Y	Y	Y	N	N	Y	N
**Justo-Alonso *et al*. (2020)**	8	Y	Y	Y	Y	Y	Y	Y	Y
**Kalaitzaki (2021)**	6	Y	Y	Y	Y	N	N	Y	Y
**Kamberi *et al*. (2020)**	4	N	Y	Y	Y	N	N	Y	N
**Karaivazoglou *et al*. (2021)**	7	N	Y	Y	Y	Y	Y	Y	Y
**Landi *et al*. (2020)**	8	Y	Y	Y	Y	Y	Y	Y	Y
**Lenzo *et al*. (2020)**	7	N	Y	Y	Y	Y	Y	Y	Y
**Lòpez-Moreno *et al*. (2020)**	5	Y	Y	Y	Y	N	N	Y	N
**Lopez Bueno *et al*. (2020a)**	7	Y	Y	Y	Y	Y	Y	N	Y
**Lopez Bueno *et al*. (2020b)**	6	N	Y	Y	Y	Y	Y	N	Y
**Lorant *et al*. (2021)**	7	N	Y	Y	Y	Y	Y	Y	Y
**Margetic *et al*. (2021)**	7	N	Y	Y	Y	Y	Y	Y	Y
**Mariani *et al*. (2020)**	6	Y	Y	Y	Y	N	N	Y	Y
**Maugeri *et al*. (2020)**	5	N	Y	Y	Y	N	N	Y	Y
**Mazza *et al*. (2020)**	8	Y	Y	Y	Y	Y	Y	Y	Y
**Méndez-Giménez *et al*. (2021)**	7	N	Y	Y	Y	Y	Y	Y	Y
**Silva Moreira *et al*. (2021)**	8	Y	Y	Y	Y	Y	Y	Y	Y
**Munk *et al*. (2020)**	5	N	Y	Y	Y	N	N	Y	Y
**Neill *et al*. (2021)**	8	Y	Y	Y	Y	Y	Y	Y	Y
**Nese *et al*. (2022)**	3	N	Y	N	N	N	N	Y	Y
**Odriozola-Gonzalez *et al*. (2022)**	8	Y	Y	Y	Y	Y	Y	Y	Y
**Orlandi *et al*. (2021)**	8	Y	Y	Y	Y	Y	Y	Y	Y
**Pakenham *et al*. (2020)**	8	Y	Y	Y	Y	Y	Y	Y	Y
**Panno *et al*. (2020)**	8	Y	Y	Y	Y	Y	Y	Y	Y
**Papandreu *et al*. (2020)**	8	Y	Y	Y	Y	Y	Y	Y	Y
**Paulino *et al*. (2021)**	8	Y	Y	Y	Y	Y	Y	Y	Y
**Perez *et al*. (2021)**	8	Y	Y	Y	Y	Y	Y	Y	Y
**Pérez-Rodrigo *et al*. (2021)**	7	N	Y	Y	Y	Y	Y	Y	Y
**Pieh *et al*. (2021)**	6	N	Y	Y	Y	N	Y	Y	Y
**Pieh *et al*. (2020)**	7	N	Y	Y	Y	Y	Y	Y	Y
**Prati (2021)**	7	N	Y	Y	Y	Y	Y	Y	Y
**Prete *et al*. (2020)**	8	Y	Y	Y	Y	Y	Y	Y	Y
**Ramiz *et al*. (2021)**	6	Y	Y	Y	Y	N	N	Y	Y
**Rania and Coppola (2021)**	7	N	Y	Y	Y	Y	Y	Y	Y
**Rettie and Daniels (2021)**	8	Y	Y	Y	Y	Y	Y	Y	Y
**Rodriguez Rey *et al*. (2020)**	8	Y	Y	Y	Y	Y	Y	Y	Y
**Rossi *et al*. (2021)**	8	Y	Y	Y	Y	Y	Y	Y	Y
**Rotărescu *et al*. (2021)**	6	Y	Y	Y	Y	N	N	Y	Y
**Saita *et al*. (2021)**	5	Y	Y	Y	Y	N	N	Y	N
**Skapinakis *et al*. (2020)**	5	N	Y	Y	Y	N	N	Y	Y
**Suso Ribeira and Martin-Burfau (2020)**	8	Y	Y	Y	Y	Y	Y	Y	Y
**Szabó *et al*. (2020)**	6	Y	Y	Y	Y	N	N	Y	Y
**Valiente *et al*. (2021)**	7	N	Y	Y	Y	Y	Y	Y	Y
**Vicario-Merino (2020)**	4	N	Y	Y	Y	N	N	Y	N/A
**Vujčić *et al*. (2021)**	8	Y	Y	Y	Y	Y	Y	Y	Y
**León-Zarceño *et al*. (2021)**	5	N	Y	Y	Y	N	N	Y	Y

**Table A3.** Critical appraisal of cohort studies

Study	Johanna Briggs Institute Score (Moola *et al*., 2020)	Were the criteria for inclusion in the sample clearly defined?	Were the study subjects and the setting described in detail?	Exposure measured in a valid and reliable way?	Objective, standard criteria used for measurement of the condition?	Confounding factors identified?	Strategies to deal with confounding factors stated?	Outcomes measured in a valid and reliable way?	Appropriate statistical analysis used?	Was the follow-up time reported and sufficient to be long enough for outcomes to occur?	Was follow-up complete, and if not, were the reasons to loss to follow up described and explored?	Were strategies to address incomplete follow up utilised?
**Ausin *et al*. (2021)**	11	Y	Y	Y	Y	Y	Y	Y	Y	Y	Y	Y
**Cheval *et al*. (2021)**	11	Y	Y	Y	Y	Y	Y	Y	Y	Y	Y	Y
**O'Connor *et al*. (2021)**	10	N	Y	Y	Y	Y	Y	Y	Y	Y	Y	Y
**Ozamiz-Extebarria *et al*. (2020)**	8	N	Y	Y	Y	N	N	Y	Y	Y	Y	Y
**Salfi *et al*. (2020)**	10	N	Y	Y	Y	Y	Y	Y	Y	Y	Y	Y
**Velotti *et al*. (2021)**	10	N	Y	Y	Y	Y	Y	Y	Y	Y	Y	Y
**Zavlis *et al*. (2021)**	11	Y	Y	Y	Y	Y	Y	Y	Y	Y	Y	Y
