# Supplementary material for: Psychological impact of the quarantine during the COVID-19 pandemic on the general European adult population: a systematic review of the evidence
Source: Epidemiol Psychiatr Sci. 2022 Apr 27;31:e27. doi: 10.1017/S2045796022000051 (PMC9069583; doi:10.1017/S2045796022000051)
Supplement: Supplementary file 1 [file S2045796022000051sup001.docx]

**Supplementary Material**

**Table 1: A brief summary of the studies included in this review.**

|  | Country | Participants (N.) | Quarantine period | Measures | Assessment tool | Main objectives |
| --- | --- | --- | --- | --- | --- | --- |
| Kamberi *et al.* 2020 | Albania | 715 | This study was carried out from 25th March – 20th April 2020, just two weeks after quarantine started | Mental disorders, especially depressive disorder | PHQ-9 | To assess the mental state of Albanian people residing in the country and abroad during the Covid-19 pandemic as well as to contribute to filling the scientific knowledge gap |
| Pieh *et al.* 2020 | Austria | 1005 | Survey started after 4 weeks of lockdown and was performed for two weeks (until April 30th) | Quality of life, well-being, depression, anxiety, stress and sleep quality | WHO Quality Of Life-BREF, WHO-5 Well-Being Index, PSS-10, PHQ-9, GAD-7, ISI | To evaluate mental health and the effect of age, gender, income, work and physical activity |
| Ellen & De Vriendt 2021 | Belgium | 1781 | During the first lockdown between April 24 and May 4, 2020 | Mental health, resilience, meaning in activities | GHQ-12, Connor-Davidson Resilience Scale, Engagement in Meaningful Activities Survey | To identify correlates of adults’ mental health and to assess the role of meaningful activities in particular |
|  |  |  |  |  |  |  |
| Lorant *et al.* 2021 | Belgium | 20,792 | The survey was open from 20 March to 9 April 2020 (it started 3 days after the beginning of the lockdown). Those data were compared with the Belgian Health Interview Survey of 2018 | Psychological distress, social support | GHQ-12, the Short Loneliness Scale, and the Oslo Social Support Scale | To investigate the risk of psychological distress  during the early days of the lockdown. To compare the results to a dataset from a pre- COVID19 national survey, helping to shed light on the changes, associated with the pandemic and the accompanying measures. To disentangle the different pathways involved and to compare the level of different symptoms in a pre-COVID19 period with the level of symptoms at the beginning of the lockdown |
| Đogaš *et al.* 2020 | Croatia | 3027 | Ten days of the lockdown (from April 25 to May 5) | Self-care, nutritional status, physical activity, sleep routines and mood changes | Online self-report questionnaire | To investigate the effect of COVID-19 lockdown on lifestyle behaviours and mood changes in the Croatian general population |

| Margetić *et al.* 2021 | Croatia | 2641 | During the COVID-19 lockdown, a few weeks after the capital was hit by the earthquake  (between April 4 and 27, 2020) | Distress, coping, personality and social support | DASS-21, the Brief COPE, the International Personality Item Pool, the Duke-UNC Functional Social Support Questionnaire | To evaluate psychological distress (stress, anxiety and depression) and personality traits, coping styles, and sociodemographic characteristics as possible predictive factors of distress |
| --- | --- | --- | --- | --- | --- | --- |
| Beck *et al.* 2021 | France | 1004 | 2 weeks after the lockdown was implemented (from March 31 to April 2) | The prevalence of sleep problems before and during the confinement and use of sleeping pills | Items assessing self-reported sleep problems (Have you been having trouble sleeping during the last 8 days?, Over the last 12 months, have you taken sleeping pills or drugs for sleep ) and a quality of life scale | To observe the prevalence of sleep problems and how people cope with trouble sleeping during this extraordinary period |
| Haesebaert *et al.* 2020 | France | 11391 | second week of the lockdown period (March 25-30) | Wellbeing and stress | Warwick Edinburgh Mental Wellbeing Scale, Visual Numerical Scales for stress | To investigate sociodemographic and environmental determinants of wellbeing |
| Ramiz *et al.* 2021 | France | 1237 | The recruitment process began in November 2014. The lockdown questionnaire was sent between April 15, 2020 and May 4, 2020. Comparisons were done with answers provided 4.8 years earlier on average. | Self-perceived mental health and self-perceived physical health, anxiety symptoms | PHQ-9, GAD-7 | To measure the consequences of the national lockdown  and to assess potential vulnerability and resilience factors. |
| Benke *et al.* 2020 | Germany | 4335 | Between 17th April and 15th May 2020, four weeks after all German federal states had implemented public health measures | Depression, Generalized anxiety, health anxiety, loneliness, psychosocial distress, general life satisfaction | PHQ-9, GAD-7; short version of the Whitely Index, the 3-item version of the UCLA Loneliness Scale, the Stress module of the PHQ, general life satisfaction was assessed with a single item | To investigate the role of sociodemographic and COVID-19 related factors for immediate mental health consequences in a nationwide community sample of adults |
| Jung *et al.* 2020 | Germany | 3545 | During the height of lockdown measures in Germany from 1 April 2020 to 15 April 2020 | Anxiety and depression, psychosocial distress, sense of coherence, sexual contentment, and sleep quality | PHQ-4, the PHQ stress module, the WHO-5Well-being Index (WHO-5), and the Sense of Coherence Scale–short form Leipzig (SOC-L9) | To systematically assess mental health in response to the pandemic and the measures taken in order to contain it. |
| Munk *et al.* 2020 | Germany | 949 | 14 days after lockdown (March 27th-April 3rd) | Symptoms regarding depression, (health) anxiety, panic disorder, obsessive-compulsive disorder, and lockdown related behaviour. Coping styles, resilience and well-being | BDI, PHQ, the German version of the Short Health Anxiety Inventory, the German version of the Obsessive-Compulsive Inventory-Revised, German version of the brief resilience scale and of the WHO-5 well-being index | To assess prevalence of mental disorders and potential behaviours/states that can have protective functions on preventing severe mental problems |
| Fontoulakis *et al.* 2021 | Greece | 3399 | The data were collected from April 11th to May 1st, 2020, during the period of the full implementation of lockdown | Distress, depression, anxiety and suicidal thoughts | CES-D and study specific questionnaire measured suicidal thoughts and emotional state | To investigate the rate of clinical depression in the adult population.  Secondary aims were to investigate the changes in anxiety, distress, suicidal ideation and their relations with a number of personal and interpersonal/social variables |
| Kalaitzaki, 2021 | Greece | 1661 | Amid the lockdown (5–30 April, 2020) | Posttraumatic symptoms, posttraumatic growth, resilience, and coping strategies | Posttraumatic Check List-5, Secondary Traumatic Stress Scale, Post-Traumatic Growth Inventory, Brief Resilience Scale, Brief Coping Orientation to Problems Experienced Inventory | To examine (a) the prevalence and severity of the psychological impact–both negative and positive–of the COVID-19 outbreak (b) identify the profile of the population subgroup, i.e., those at higher risk and those that manage to adapt and grow, and (c) examine the personal resources of resilience and coping responses used by different population subgroups |
| Karaivazoglou *et al.* 2021 | Greece | 1443 | Between 10 April and 4 May 2020, period during which the whole country was under strict lockdown measures | Post-traumatic stress symptoms and psychological functioning | IES-R, HADS | To determine the prevalence of psychological distress and post-traumatic stress symptoms in the Greek population during the first COVID-19 lockdown, and to detect potential correlates |
| Skapinakis *et al.* 2020 | Greece | 3379 | Peak of COVID-19 lockdown (8th-12th of April) | depression and anxiety symptoms, coping strategies, illness beliefs | PHQ-9, GAD-7, Brief COPE questionnaire, Illness Perception Questionnaire- Revised | To investigate the prevalence of anxiety and depression, the emotional impact of the epidemic and the effect of coping strategies and illness perceptions in mental health |
| Szabò *et al.* 2020 | Hungary | 431 | In COVID-19 lockdown between 7–20 May 2020 | Perceived stress, levels of anxiety and depression, self- reported health, number of complaints, strategies in coping with stressful situations | PSS-10, GAD-2; PHQ-2; European Quality of Life Visual Analogue Scale (EQ-VAS); Self-administered inventory of complaints (Hungarian questionnaire); Shortened (Hungarian) version of the Ways of Coping Questionnaire; 2 open-ended questions to examine the participants’ mood and ways of coping during the pandemic | To explore certain psychological effects of the coronavirus pandemic on Hungarian adults |
| Bacaro *et al.* 2020 | Italy | 1989 | The survey was distributed from 1st April to 4th May 2020 | Insomnia severity, depression, anxiety, sleep hygiene behaviours, emotion regulation, cognitive flexibility, perceived stress, health habits, self-report of mental disorders, and variables related to individual differences in life changes due to the pandemic’s outbreak | ISI, Emotion Regulation Questionnaire, PSS-10, HADS | To provide a snapshot of the prevalence of insomnia disorder in the Italian population and investigate its association with potential risk factors and its link with anxiety and depression symptoms |
| Balsamo & Carlucci 2020 | Italy | 3672 | From March 21 to 26, immediately after the nationwide lockdown issued by the Italian Government on March 11 (#iorestoacasa) | Depression, anxiety, worry about being infected with COVID-19 | Teate depression inventory, State-Trait Inventory for Cognitive  and Somatic Anxiety | To explore (1) the likely effects of quarantine on mental health (2) the factors that contribute to, or mitigate, these consequences |
| Bonati *et al.* 2021 | Italy | 20 518 | From 6 to 20 April 2020 | Distress, depression, anxiety and physical symptoms | CPDI | To collect information on psychologically distressing experiences of Italians living in quarantine during the COVID-19 pandemic |
| Cancello *et al.* 2020 | Italy | 490 | April 15- 4 May, 2020 | Changes during lockdown (weight, sleep quality, physical activity, cigarette consumption, appetite, food purchase and intake, quality of the diet, and use of supplements) | The questionnaire included 31 questions on baseline data and changes during lockdown | To observe the effects of the lockdown on the reported lifestyle habits changes. To give a picture of the perception of changes that occurred in the main lifestyle components and to identify their determinants |
| Casagrande *et al.* 2020 | Italy | 2291 | Study began 10 days after the beginning of quarantine due to the COVID-19 (March 18- April 2) | Anxiety, general psychological wellbeing or distress, sleep quality and specific symptoms concerning the COVID-19 emergency, similar to PTSD symptoms | GAD-7, Psychological General Well-Being Questionnaire, Pittsburgh Sleep Quality Index and a modified version of PTSD Checklist for DSM-5 | To briefly look into the psychological cost of the COVID-19 emergency. Specifically, sleep quality, generalized anxiety, psychological distress, and the risk of PTSD were investigated |
| Casali *et al.* 2021 | Italy | 944 | One month after lockdown began | Character strengths, depression, anxiety, stress, general psychological health and COVID related self-efficacy | Values in Action Inventory of Strengths (VIA-IS- 120), DASS-21, GHQ-12, Self-efficacy measure for COVID-19 (SEC) | To analyse whether and which character strengths are associated with people's mental health and COVID related self-efficacy |
| Castellini *et al.* 2021 | Italy | 671 subjects: 130 participants with both pre-lockdown (T0) and in-lockdown (T1) evaluations, and 541 additional subjects recruited at T1 | A few weeks before the first cases of COVID- 19 (T0: from December 1st, 2019 until January 15th, 2020) and six weeks after the Italian Government declaration of lockdown (T1: from April 22nd, 2020 until May 3rd, 2020) | General psychopathology | The Brief Symptom Inventory, IES-R | To investigate general psychopathology a few weeks before the COVID-19 outbreak (T0) and during lockdown (T1), and the associations between lockdown-related environmental conditions, self-perceived worsening in daily living and psychopathology |
| Cecchetto *et al.* 2021 | Italy | 365 | During the second week of Phase 2, from the 14th of May to the May 19, 2020 | Self-reported physical (BMI), psychological (Alexithymia), affective (anxiety, stress, and depression) and social (income, workload) state | The 7-Item Binge-Eating Disorder Screener, Toronto Alexithymia Scale, The Dutch Eating Behavior Questionnaire, GAD-2, PHQ-2, PSS-10 | To investigate the impact of lockdown on emotional and binge eating |
| Cerami *et al.* 2020 | Italy | 1163 | March 14 and March 21, 2020 | Perceived impact of COVID-19 outbreak, depression, anxiety, stress, loneliness and coping strategies | The perceived impact of COVID-19 outbreak was assessed with 4 items for health and 4 items for economy. DASS-21, Italian Loneliness Scale, Italian version of the Coping Orientation to the Problems Experienced ( COPE-NVI-25) | To assess the influence of psychosocial variables on individual differences from the perceived impact of the outbreak on the issues of health and economy |
| Cerbara *et al.* 2020 | Italy | 140656 | Data collection started 11 days after the beginning of lockdown (March 22 to April 2) | basic emotions (happiness, sadness, fear, anxiety) and resilience | A simple question was asked in order to investigate the intensity of the respondents' emotions. | To explore the reasons and the ways through which social distancing generates negative emotions in individuals who experienced the lockdown. To check the existence of relations between negative emotions and the satisfaction of basic needs |
| Conversano *et al.* 2020 | Italy | 6412 | Survey was launched on March 13, 2020 and closed April 6, 2020 | Psychological distress and mindfulness disposition | The Italian version of the Symptoms Checklist-90 and the Mindfulness Attention Awareness Scale | To identify individuals at higher risk for psychological distress while measuring the weight of mindfulness disposition in protecting their mental health and to assess the relationship between mindfulness and several psychiatric symptoms of distress |
| Coppola *et al.* 2021 | Italy | 1250 | The research lasted 10 days and was carried out after the first 2 weeks of lockdown | Spiritual well-being, mental health | Jarel Spiritual Well-Being Scale, GHQ-12 | To investigate how spiritual well-being dimension was faced by Italian adults during the lockdown subsequent to the COVID-19 outbreak. To explore psychological mental health in terms of the psychological impact of the pandemic and to analyse if there were differences in the perception of spiritual well-being and mental health compared to the pre-pandemic data in the general population |
| Costantini & Mazzotti 2020 | Italy | 329 | Phase 1 of the Pandemic period, from April 15th to April 24th | Peritraumatic distress, degree of emotional impact of the pandemic and the presence of probable post-traumatic disorder | CPDI and IES-R | The validation of the Italian version of the CPDI and the measurement of the prevalence of peritraumatic distress |
| Di Corrado *et al.* 2020 | Italy | 670 | Initial stage of the outbreak: first two weeks of restrictive procedures | Attitudes towards distressing situations in general, the valuation of positive and negative emotions regarding the present situation and the degree of experienced symptoms, habits regarding physical activity | The survey was created ad hoc for the purposes of the study. | To survey the general community to better recognise their levels of psychological impact, emotional responses and maintenance **of** their daily exercise or physical activity routines |
| Di Crosta *et al.* 2020 | Italy | 1253 | Peak period of the contagion in Italy (April 1 to April 20), three weeks after national lockdown started | Fear for COVID, personality traits, post-traumatic symptoms | Fear for COVID (ad hoc Questionnaire), the Big Five Inventory-10 Item, IES-R | To report the prevalence of high psychological distress, especially considering that this aspect is consistently associated with PTSD symptoms. To identify the risk factors for high PTSD symptoms |
| Di Giuseppe *et al.* 2020 | Italy | 5683 | Data were collected from March 13 to March 18, within the first week of lockdown | Psychological distress, post-traumatic symptoms, and defence mechanisms | Symptom Checklist-90 (SCL-90), IES-R, and the Defense Mechanisms Rating Scale-Self-Report-30 (DMRS-SR-30) | To investigate the psychological impact of the coronavirus disease 2019 crisis and the role of defence mechanisms as protective factors against distress |
| Di Renzo *et al.* 2020 | Italy | 602 | Last four weeks of COVID-19 quarantine and home confinement (24 April-18 May, 2020) | Anxiety symptoms, depression and signs of addiction towards certain types of foods | Hamilton Anxiety Rating Scale, Hamilton Depression scale and Yale Food Addiction Scale | To analyse the psychological status during the COVID-19 pandemic and its correlation with the eating habits |
| Favieri *et al.* 2021 | Italy | 1639 | Data refers to the period from March 18 to 25, 2020 | Subjective general psychological well-being, specific symptoms consequent to the COVID-19, similar to PTSD symptoms | The General Psychological Well-Being Index and a modified version of The Post-Traumatic Stress Disorder Related to COVID-19 (COVID-19-PTSD) | To provide a photograph of the Italian condition in the first weeks of the restrictive measures related to the period immediately following the promulgation of the “I stay home” decree determined by the broad and severe diffusion of the COVID-19 in Italy |
| Ferrucci *et al.* 2020 | Italy | 10025 | From 4 to 18 March 2020 (from Week 2 -4 of the outbreak) | Self-reported psychological impact | An ad hoc questionnaire was designed. The entire survey was comprised of 21 closed questions | To assess the immediate psychological impact of the COVID-19 epidemic on emotional health and well-being |
| Fiorenzato *et al.* 2021 | Italy | 1215 | The end of a seven to ten -week imposed lockdown and home confinement (from April 29 to May 17, 2020) | Depression, anxiety, Sleep, appetite, libido and hypochondria changes | HADS, BDI-II | To explore subjective cognitive functioning and mental health changes and their possible interplay related to COVID-19-lockdown. To investigate potential risk factors to identify more vulnerable groups |
| Fiorillo *et al.* 2020 | Italy | 20720 | Different weeks in "phase 1" between March and May (it was officially launched on March 30, 2020 and ended on May 4) | The severity of depression, anxiety and stress symptoms. The levels of global mental health status, of obsessive-compulsive and post-traumatic symptoms, presence of severity and insomnia, the levels of perceived loneliness and the presence of suicidal ideation/thoughts. Exploratory variables include coping strategies, levels of post-traumatic growth, perceived social support and resilience | DASS-21, GHQ-12, The Obsessive-Compulsive Inventory-Revised version (OCI-R), The Suicidal Ideation Attributes Scale (SIDAS), The Severity of Acute Stress Scale Symptoms Adult scale (SASS), The Impact of Events Scale (IES), The UCLA loneliness scale, The brief-COPE, the short form of Post-Traumatic Growth Inventory (PTGI), the Connor Davidson Resilience scale (CD-RISC), the Multidimensional Scale of Perceived Social Support (MSPSS), The Maslach Burnout Inventory (MBI) | To evaluate the impact of the lockdown on mental health (to explore the levels of depressive, anxiety and stress symptoms during the different weeks of the lockdown). To identify possible risk and protective factors for mental health outcome |
| Franceschini *et al.* 2020 | Italy | 6519 | During the whole of the COVID-19 lockdown (from March 10–1st phase to May 4–2nd phase) | Sleep quality, mental health | Medical Outcomes Study–sleep scale (MOS-SS) and the short form of DASS –21 | To study the quality of sleep and its connection to distress levels and to evaluate how lifestyle changed during the lockdown |
| Gualano *et al.* 2020 | Italy | 1515 | Last 14 days of the Italian lockdown (April 19th-May 3rd) | Depression screening, anxiety disorders screening and sleep disturbances | PHQ-2 , GAD-2, ISI | To estimate the psychological impact of COVID-19 and related restrictive measures and to evaluate the prevalence of depressive symptoms, anxiety symptoms and sleeping issues |
| Landi *et al.* 2020 | Italy | 944 | not specified, length unclear. The mean number of days in lockdown is 35 | psychological flexibility, trait health anxiety, COVID-19 distress, anxiety and depression | The Short Health Anxiety Inventory (SHAI), The Multidimensional Psychological Flexibility Inventory (MPFI), CPDI, PHQ-9 | To explore the role of psychological flexibility in protecting people at risk of poorer mental health impacts due to elevated health anxiety |
| Lenzo *et al.* 2020 | Italy | 6314 | Participants were recruited between March 29 and May 04 2020 | depression, anxiety, and stress and resilience | DASS-21 and the Resilience Scale (RS) | To examine depression, anxiety, and stress among the Italian general population during the phase characterized by lockdown, and to investigate the role of resilience as a potential predictor |
| Mariani *et al.* 2020 | Italy | 96 | 3 weeks after the imposition of the lockdown restrictions | Psychological and psychosomatic symptoms, coping and perceived social support. | Coping Inventory for Stressful Situations (CISS), Multidimensional Scale of Perceived Social Support (MSPSS) and Symptom Checklist-90-Revised (SCL-90-R) | To explore the effect of coping strategies and perceived social support on depressive and anxious symptomatology during the COVID-19 pandemic |
| Maugeri *et al.* 2020 | Italy | 2524 | Period of 30 days (from April 1st to April 30th), three weeks after the beginning of lockdown measures | Physical activity energy expenditure before and during quarantine and psychological wellbeing | International Physical Activity Questionnaire and Psychological General Well Being Index | To examine changes in the physical activity levels during self-quarantine and the impact of exercise on psychological health |
| Mazza *et al.* 2020 | Italy | 2766 | Study began 10 days after the beginning of Italian quarantine (18-22 March) | Emotional states of depression, anxiety and stress, personality trait domains | DASS-21, Personality Inventory for DSM-5 Brief Form Adult | To survey psychological distress, with the aim of establishing the prevalence of psychiatric symptoms and identifying risk and protective factors for psychological distress among sociodemographic and personality variables |
| Nese *et al.* 2020 | Italy | 931 | (29 March to April 4) | Self-reported compliance with containment measures over time, three hypothetical risk levels, perceived risk, generalized anxiety, intolerance of uncertainty and relevance of several psychological needs whose satisfaction is precluded at the survey time | The compliance with containment measures was quantified using a questionnaire inspired by the Medical Decision Making Questionnaire (MDMQ, Bruce et al., 2016). Perceived risk was assessed by asking the subjects to subjectively predict the likelihood of contracting COVID-19 in their living area. A set of questions concerning five proposed psychological needs that may be no longer satisfied because of the containment measures was presented (meeting partner/friends/going to work as usual/ doing outdoor sports/ engaging in recreational activities in public places). GAD-7 and Intolerance of Uncertainty Scale, short form (IUS-12) | To explore the self-reported future compliance of citizens with such measures and its relationship with potentially impactful psychological variables (perceived risk, anxiety, intolerance of uncertainty, psychological needs) |
| Orlandi *et al.* 2021 | Italy | 2218 | Data were collected during the fifth week of the home confinement | Physical activity levels and mental status and psychosomatic disorders | the short version of IPAQ, four subscales of the short form of the SF-36 Health Survey Version 1 questionnaire | To assess gender differences in the impact of lockdown on physical activity and lifestyle habits |
| Pakenham *et al.* 2020 | Italy | 1035 | not specified, length unclear. The mean number of days in lockdown is 39 | psychological flexibility and inflexibility, peritraumatic distress, anxiety, depression | The Multidimensional Psychological Flexibility Inventory (MPFI), CPDI, GAD-7, PHQ-9 | To investigate the roles of psychological flexibility and inflexibility in moderating the effects of COVID-19 risk factors on three mental health outcomes: COVID-19 peritraumatic distress, anxiety, depression |
| Panno *et al.* 2020 | Italy | 1519 | During the Italian lockdown (9 March 2020 - 4 May 2020) | COVID-19 related distress, alcohol problems, addiction-like symptoms in relation to excessive and compulsive social media use, food addiction | IES-R, CAGE questionnaire, Bergen Social Media Addiction Scale (BSMAS), Yale Food Addiction Scale (YFAS) | To investigate the association between COVID-19 related distress and: alcohol problems, social media addiction symptoms, food addiction symptoms |
| Prati 2021 | Italy | 1569 | April 2020 | mental health symptoms, well-being, worry about the epidemic of COVID-19, likelihood of infection, coping | The Italian version of the Mental Health Continuum–Short Form, GHQ-12 | To investigate the psychological impact of national quarantine and the psychosocial factors that may influence this impact |
| Prete *et al.* 2020 | Italy | 618 | Between March 26 and April 8 | Levels of anxiety, psychological reactions to dangerous events, affective worry and protective behaviours | The Italian version of the State-Trait Anxiety Inventory, The Pre-traumatic stress reaction Check List | To investigate whether protective behaviours might have been enhanced or limited by anxiety and emotional reactions to previous experience of stressful conditions |
| Rania & Coppola 2021 | Italy | 721 | The survey was carried out over a 10-day period, after the first 2 weeks of lockdown, from March 25th to April 4th, 2020 | Subjective global happiness, the severity of mental problems, loneliness | Subjective Happiness Scale, GHQ-12, the Three-Item Loneliness Scale | To analyse the perception of happiness, mental health, and the sense of loneliness experienced by adults during the lockdown due to the COVID pandemic |
| Rossi *et al.* 2021 | Italy | 21334 | At 3 weeks after the beginning of the lockdown | Depressive and anxiety symptoms, resilience and perceived stress | PHQ-9, GAD-7, PSS-10.  Resilience was measured using the Italian version of the Resilience Scale for Adults (RSA) | To explore the role of resilience as a mediator between stressful COVID-19 related life events and depressive and, anxiety symptoms and perceived stress, and to ascertain the role of age as a moderator of the mediator’s effect |
| Saita *et al.* 2021 | Italy | 319 | Data were collected from 13 April to 10 May 2020 | Anxiety, depression, and perceived stress | GAD-7, PHQ-9 and the Perceived Stress Scale | To investigate the impact of the pandemic and the subsequent restrictive measures on the psychological health of Italian men and women living in Lombardy, one of the worst-hit regions. The study also aimed at identifying what factors are associated with specific psychological outcomes |
| Cellini *et al.* 2021 | Italy and Belgium | 2272 participants, 1622 Italians and 650 Belgian | In both countries, participants completed the survey, from April 1st to May 19th, 2020 | Sleep timing and quality | Pittsburgh Sleep Quality Index | To investigate how the COVID-19-related restrictions affected people's self-reported sleep timing and sleep characteristics |
| Bonsaksen *et al.* 2020 | Norway | 4527 | Between 8 April 2020 and 20 May 2020 | Current post-traumatic stress symptoms | The PTSD Checklist for DSM-5 (PCL-5) | To examine the prevalence of current symptom-defined PTSD and associated factors in the Norwegian population |
| Bodecka *et al.* 2021 | Poland | 230 | Context of lockdown due to COVID-19 epidemic | Depressive symptoms, perceived stress and present hedonistic time perspectives | PHQ-9, PSS-10, the Zimbardo Time Perspective Inventory | To explore gender differences in the relationships between Present Hedonistic Time perspective and depressive symptoms or perceived stress during COVID-19 lockdown |
| Bartoszek *et al.* 2020 | Poland | 471 | 1.5 weeks after home confinement restrictions were introduced in Poland (3 April 2020). The questionnaire was available for the following two weeks | Insomnia, depression, feelings of loneliness and physical, mental and social fatigue | ISI, BDI, Revised University of California, Los Angeles (R-UCLA) Loneliness Scale, and Daily Life Fatigue scale (DLF) | To measure indicators of mental well-being in a Polish sample with regard to selected sociodemographic and health behaviour data during home confinement |
| Chodkiewicz *et al.* 2020 | Poland | 443 | April 10-20, when the lockdown had been in effect for one month | Alcohol use disorder, somatic symptoms, anxiety and insomnia, dysfunction in everyday life, and depression symptoms, stress levels and coping strategies | The Alcohol Use Disorder Identification Test (AUDIT), General Health Questionnaire (GHQ-28), PSS-10, and the Brief COPE Inventory (Mini COPE) | To survey alcohol drinking throughout the pandemic so as to investigate those factors considered most relevant; i.e., sociodemographic and clinical |
| Gambin *et al.* 2021 | Poland | 1115 | The study was conducted between 4 and 8 of May 2020. At that time, two months had passed since the detection of the first case of coronavirus infection in Poland and six weeks since the beginning of the lockdown | Anxiety and depressive symptoms, the subjective risk of: (i) COVID-19 infection; (ii) serious adverse health effects and complications due to a coronavirus infection; and (iii) threat to life as a result of an infection, possible difficulties during the pandemic and social support | PHQ-9, GAD-7, a Scale of Perceived Health and Life Risk of COVID-19 and a Scale of Pandemic-Related Difficulties | To investigate: (i) differences in levels of depressive and generalized anxiety symptoms in four age groups; (ii) differences in perceived difficulties related to the pandemic in these groups; and (iii) which factors and difficulties related to the pandemic are the best predictors of generalised anxiety and depressive symptoms in various age groups |
| Izdebsky & Mazur 2021 | Poland | 3000 | The survey was carried out online at the turn of May and June 2020 | The feeling of loneliness, periods of feeling low, experiencing depression, or poor well-being, periods of prolonged fatigue, weakness, somnolence, and concentration difficulty, tantrums, attacks of aggression, and frustration | Ad hoc questionnaire | To analyse the relationship between the occupational activity of Poles and the frequency of occurrence of the symptoms of worsened mental health during the first COVID-19 lockdown |
| Antunes *et al.* 2020 | Portugal | 1404 | 1-15 April, during which a state of emergency was decreed and social confinement imposed | Lifestyle habits (sleeping habits, eating habits, physical activity habits), anxiety levels and satisfaction of basic psychological needs | They were asked to self-report on sleep duration and quality, the amount and frequency of food intake and time spent watching pandemic related news. The International Physical Activity Questionnaire, the Basic Need General Satisfaction Scale and the State-Trait Anxiety Inventory | To characterise the lifestyle habits, anxiety levels and basic psychological needs, including a comparison between genders and group ages |
| Paulino *et al.* 2021 | Portugal | 10 529 | 24-27 March, one week after the state of emergency was declared | Depression, anxiety and stress | IES-R, DASS-21, | To evaluate the immediate psychological impact, as well as anxiety, depression and stress |
| Silva Moreira *et al.* 2021 | Portugal | 1280 | The survey began on 23 March 2020, four days after the declaration of the emergency state by the Portuguese government. Data were collected until the end of March | Anxiety, depression, stress and obsessive-compulsive symptoms | DASS-21, The Obsessive-Compulsive Inventory—Revised scale (OCI-R) | To comprehensively characterise a set of demographic, social and mental health variables in a Portuguese sample during the outbreak |
| Rotărescu *et al.* 2021 | Romania | 495 | Between April and May 2020, during the strict isolation period imposed by the national government | Trait and state anxiety, resilience measurement, experiential avoidance/psychological flexibility, family relationships | The State-Trait Anxiety Inventory, The Acceptance and Action Questionnaire 2, The Aggression Questionnaire, The Family Connectedness Questionnaire, and Connor-Davidson Resilience Scale 10 | To identify the impact of the environment on the level of anxiety. The influence of protective factors, such as resilience, on the anxiety level, could vary in stressful situations, depending on gender |
| Vujcˇic´ *et al.* 2021 | Serbia | 1057 | Data collection took place from 23 March 2020 (8 days after declaring the state of emergency in Serbia) until 25 April 2020 (still in a state of emergency) | Depression, anxiety, and stress | DASS-21 | To determine the impact of the COVID-19 epidemic on the mental health of the Serbian population and to identify associated factors during the state of emergency and lockdown |
| Balanzá-Martínez *et al.* 2021 | Spain | 1254 | The survey was initiated one month after lockdown was issued (April 15- 22). Results refer to the first week of data collection | Changes in seven lifestyle domains (diet and nutrition, substance abuse, physical activity, stress management, restorative sleep, social support and environmental exposures), current depression and anxiety, alcohol abuse | The Short Multidimensional Inventory Lifestyle Evaluation (SMILE-C), PHQ-2, GAD-7, AUDIT alcohol consumption questions (AUDIT-C) | To describe self-reported changes in lifestyle behaviours and to evaluate the psychometric properties of the Short Multidimensional Inventory Lifestyle Evaluation (SMILE). To evaluate factors associated with lifestyle scores |
| Carriedo *et al.* 2020 | Spain | 1795 | Seven days after the mandatory shelter in place health order was issued (March 21st) | Resilience, physical activity, BMI before and after the first week of confinement | The Connor-Davidson resilience scale (CD-RIS), International Physical Activity Questionnaire (IPAQ) | To examine the impact of COVID-19 forced isolation on citizens' resilience, as well as the connections between resilience, physical activity, gender, weight and BMI before and after confinement |
| García-Álvarez *et al.* 2020 | Spain | 21207 | Between 19 and 26 March, five days after the official declaration of alarm and the lockdown order | Depression, anxiety, stress and current subjective distress | DASS-21, and IES | To describe the early psychological impact of COVID-19 and its contributing factors, globally and according to mental status |
| Gismero- González *et al.* 2020 | Spain | 906 | Data were collated between May 8 and May 13, when the lockdown enacted by the Spanish government due to the COVID-19 health crisis had been in force for 8 weeks | Physical Health and Health Habit Variables, mood variables, resilience | Positive and Negative Affect Schedule (PANAS) Participants were asked three questions regarding, “How would you rate your overall mood before COVID-19/during lockdown/when restrictions started to be lifted”.  Spanish version of the Brief Resilience Scale (BRS) | To investigate the effects of COVID-19 quarantine on the emotional functioning of confined individuals. The possible associations between changes in emotional functioning and demographic variables, health habits, social support, and resilience were also analysed |
| Gomez-Salgado *et al.* 2020 | Spain | 4180 | Between March 26 (13 days after the start of  confinement) and April 26 | Psychological distress | GHQ-12 | To analyse the psychological distress, identifying the predictive character and role that sociodemographic variables, the presence of physical symptoms, and other health-related variables may have. The hypothesis that the health crisis caused by COVID-19 does not generate psychological distress in the population was stated |
| Günther Bel *et al.* 2020 | Spain | 407 | Weeks 2 and 3 of state-regulated lockdown (March 24 and April 7) | Psychological distress (anxiety as both a state and a trait, depression) and relationship functioning | State Trait Anxiety Inventory, BDI, Dyadic Adjustment Scale (no children in the household) or Basic Family Relations Evaluation Questionnaire | To explore the individual and relational well-being of people confined together with their partners and/or children. To compare lockdown responses from the pandemic convenience sample to benchmarks for established measures of individual, couple and parental functioning. To explore changes in relationship functioning over time and describe the ways in which participants felt their couple and family relationship had improved and/or deteriorated |
| Gutiérrez-Hernández  *et al.* 2021 | Spain | 917 | From 14 April 2020 to 21 April 2020 (lockdown was imposed on 13 March 2020 and lifted on 15 May 2020, so the study took place approximately halfway through) | Anxiety, depression, and stress levels, self-compassion, perceived infectability and germ aversion | DASS-21, the Self-Compassion Scale (SCS) and the Perceived Vulnerability to Disease Questionnaire | To assess emotional distress, measuring anxiety, depression, and stress levels. To explore the relationship between sociodemographic, clinical, and psychological variables on the one hand, and the level of emotional distress, on the other |
| Jacques- Aviñó *et al.* 2020 | Spain | 7053 | From 8 April to 28 May 2020, data collection was stopped when lockdown de-escalation  started | Anxiety and depression | GAD-7 and PHQ-9 | To assess the relationship between social impact and mental health during COVID-19 lockdown measures, taking a gender-based approach into account |
| Janè-Llopis *et al.* 2021 | Spain | 37810 | from 21 April to 20 May 2020, 5 weeks after confinement had been imposed | Depression, anxiety, well-being, alcohol | PHQ-8, the GAD-7, the Short Warwick–Edinburgh Mental Well-being Scale, the Alcohol Use Disorders Identification Test | To address the impact of confinement on mental ill-health and addictions, address the link between reported length of confinement and severity, and provide a detailed unpacking of the associated risk and protective factors that can provide insight for intervention preparedness in view of forthcoming partial or total confinements |
| Jiménez *et al.* 2020 | Spain | 412 | first stage of the confinement: from 4 April to 1 May | Symptomatology of post- traumatic stress and anxiety and depression, practice of some type of meditation or self-compassion | DASS-21, IES and the Self-Compassion Scale-short form | To evaluate the psychological impact of confinement, considering any protective factors, such as the practice of meditation or self-compassion, and their relationship with different lifestyles and circumstances |
| Justo -Alonso *et al.* 2020 | Spain | 3524 | Data were collected from March 23 to March 28, 2020 (9 to 14 days after the declaration of the state of emergency) | Depression, anxiety and stress, psychological impact | DASS-21 and IES-R | To explore, in specific age ranges, the mental health state of a Spanish community sample in the early stages of the pandemic |
| Lopez Bueno *et al.* 2020a | Spain | 2250 | From the seventh day of enacted national confinement (22-29 March) | Health habits (physical activity, alcohol consumption, smoking, screen exposure and sleep hours) and perceived anxiety and mood | Physical Activity Vita Sign (PAVS) short form, single item question to assess both current perceived anxiety and mood (how do you assess your anxiety level/mood during COVID-19 confinement?) | To analyse the association between current physical activity and current, perceived anxiety and mood |
| Lopez Bueno *et al.* 2020b | Spain | 1421 | First three weeks of confinement (March 22 and April 5). Study began the seventh day of national confinement | Health risk behaviours (exposure to screens, sleep time, physical activity, fruit and vegetable consumption, alcohol consumption and smoking habit) | The outcome variable was estimated through a set of questions concerning six heath related behaviours (Example: " What is your average daily number of hours exposed to screens such as TV, cell phone and tablet during COVID-19 confinement?). Physical Activity was estimated using the Physical Activity Vital Sign (PAVS) short version | To analyse the association between time course and health risk behaviours. |
| Lòpez-Moreno *et al.* 2020 | Spain | 675 | The study was conducted in the month before lockdown finished, (from 28 May to 21 June) | BMI, food habits, Smoking frequency Dietary supplements consumption, Sleep hours before and during lockdown and sleep quality, Exercise before and during the lockdown and mood changes | Emotional eater questionnaire and ad hoc questions | To evaluate the effects of COVID-19 home confinement on the food habits, lifestyle and emotional balance of the Spanish population |
| Méndez-Giménez *et al.* 2021 | Spain | 4811 | During the confinement phase due to the coronavirus pandemic from March 19, 2020, to April 18, 2020 (end of the first phase of the alarm state) | Depressive symptoms, physical activity | The Spanish version of the Six item self-reported scale developed by Kandel and Davies, the International Physical Activity Questionnaire (IPAQ) | To examine dose-response relationships between Physical Activity components (volume, intensity, frequency, duration) and depressive symptoms and to identify the optimal levels of PA to mitigate notable depressive symptoms |
| Odriozola-González *et al.* 2020 | Spain | 3550 | The survey was launched on, and remained open until, April 4th (8 days) | Symptoms of anxiety, depression, and stress, symptoms of posttraumatic stress disorder | DASS-21, IES | To analyse the psychological symptoms on the confined population in Spain during the outbreak of the COVID-19 crisis in relationship with the lockdown measures |
| Pèrez *et al.* 2021 | Spain | 1781 | From April 1, 2020, (18 days after the declaration of the nationwide State of Alarm and the subsequent national lockdown) to April 17 | Psychological distress, emotion regulation, positive and negative affect, sleep quality | The Brief Symptom Inventory 18 (BSI-18), Emotional Regulation Questionnaire (ERQ), The Positive and Negative Affect Schedule (PANAS), ISI | To detect levels of symptoms of psychological distress in Spanish adults during the confinement; second, to explore differences in distress and other psychological variables, depending on sociodemographic variables or history of psychopathology; and three, to explore which of the assessed variables are more strongly associated with distress |
| Pérez-Rodrigo *et al.* 2021 | Spain | 1155 | Five weeks after the start of lockdown, between 21 April and 8 May 2020 (weeks 6–8 of confinement), before the start of the de-escalation phase in Spain | Diet, usual physical activity (PA) and supplement use, dietary changes, sedentary time, PA, sleep quality, and smoking | Semi-quantitative short food frequency questionnaire (FFQ), ad hoc questionnaire inquired about practice of moderate to vigorous PA (at least 30 min) during lockdown and about the time dedicated to daily sedentary activities during confinement | To identify patterns of change in eating habits and physical activity and to identify associations with sociodemographic factors and usual habits |
| Rodriguez Rey *et al.* 2020 | Spain | 3055 | Data collection period comprised between the 17th of March ( 2 days after the state of alarm in Spain) and the 24th of March | Psychological impact of the pandemic, anxiety, stress and depression levels | IES-R, DASS-21, Participants were asked about the degree to which they were concerned about different conditions and whether they perceived the current crisis as severe | To explore the mental health status, in terms of psychological impact, caused by the pandemic. To examine the extent to which some variables are associated with psychological impact, anxiety, stress and depression |
| Suso-Ribera & Martín-Brufau 2020 | Spain | 2683 | Survey started the first day of mandatory quarantine (15 March 2020) until three days later (17 March) | Mood, quarantine-related behaviours, and personality/cognitive factors, frustration intolerance | Profile of Mood States (POMS), Emotional Intolerance Scale, Ten Item Personality Inventory | To investigate the emotional impact as well as the extent to which a comprehensive set of recommended lifestyles and socio-demographic and personality variables correlate with well-being |
| Valiente *et al.* 2021 | Spain | 1951 | The study was launched on April 8th, 26 days after the national confinement, and lasted until the 13th April 2020 | Anxiety and Economic Threat related to COVID–19, Increased substances-use, loneliness, anxiety, distress and well being | The Three item Loneliness Scale, GAD-7, The Pemberton Happiness Index | To (a) evaluate the levels of anxiety, depression and well-being in a large Spanish sample during the confinement, (b) identify potential predictor variables associated with experiencing both clinical levels of distress and well-being in a sample of 2,122 people |
| Vicario merino & Munoz-Agustin, 2020 | Spain | 144 | The Questionnaire was sent for the first time on the 22nd of March ( 8 days after the declaration of the state of alarm- until 7th of April) | Stress, anxiety and healthy habits | Inventory of anxiety situations and responses (ISRA), PSS-10 | To identify the perceived levels of stress and anxiety, identify whether there is an evolution in time, and provide health education messages |
| Leon-Zarceno *et al.* 2021 | Spain | 457 | During the days from 6 to 20 April, the time when, in Spain, confinement was in place and the highest peak of deaths and infections from COVID-19 occurred | Psychological wellbeing, in coping, in daily habits and difficulties (ad hoc questionnaire) and the level of physical activity | Spanish adaptation of psychological well-being, the Spanish adaptation of the COPE Inventory | To analyse the impact of confinement on psychological health taking into account gender and perceived changes in physical activity |
| Papandreu *et al.* 2020 | Spain and Greece | 1002 responders in Spain and 839 in Greece | This survey was launched during the last week of April in Spain (April 23rd) and the first week of May (May 3rd) in Greece, approximately 1.5-2 month after the initiation of social confinement measures in each country, and remained open until the 18th and 20th May, respectively | Eating behaviours, depressive and anxiety symptoms | The Dutch Eating Behaviour Questionnaire, PHQ-9, GAD-7 | To evaluate eating behaviours and symptoms of depression and anxiety in Spain, which experienced greater severity of the pandemic and stricter lockdown measures, compared to another Mediterranean country (Greece) with a lighter lockdown and a less severe pandemic |
| Coulthard *et al.* 2021 | United Kingdom | 620 | Early part of the lockdown (April–May 2020) | Eating behaviours, coping strategies, health anxiety and food insecurity | The Three-Factor Eating Questionnaire – Revised 18-item version, The Brief Coping Inventory, The Short Health Anxiety Inventory, The Food Insecurity Experience Scale | To examine self-reported changes in eating patterns and behaviour and associations with BMI, demographic variables, eating styles, health anxiety, food insecurity and coping strategies |
| Dawson & Golijani-Moghaddam, 2020 | United Kingdom | 555 | Since May 13th the lockdown measures started to ease slightly. period of lockdown. (15-21 May) | Psychological flexibility, the emotional, cognitive, functional and social components of mental wellbeing, depressed mood and symptoms of generalised anxiety | CompACT-8 (8 item abbreviated version of the Comprehensive Assessment of Acceptance and Commitment Therapy Process), The Short Warwick-Edinburgh Mental Well-Being Scale, PHQ-9 and GAD-7 | To examine the impact of COVID-19 on psychological health and well-being and the specific role of psychological flexibility as a potential mitigating effect |
| Groarke *et al.* 2020 | United Kingdom | 2511 | Study began on the day the UK lockdown was announced (March 23rd) and closed on April 24th 2020 | Loneliness, PTSD symptoms, Anxiety, depression and difficulties in emotion regulation. Sleep quality in general, as well as sleep quality over the previous month in relation to COVID-19, were assessed | Three-Item Loneliness Scale, PTSD Checklist for DSM-5 (PCL-5), GAD-7, PHQ-9, Difficulties in Emotion Regulation Scale-Short Form (DERS-SF) | To determine the rate of loneliness and to identify differences in sociodemographic, social, health, and COVID- 19-specific factors between people with and without loneliness to determine the risk and protective factors for loneliness |
| Jacob *et al.* 2020 | United Kingdom | 902 | The study was launched on March 17, 2020, 17 days after the first case of COVID-19 was diagnosed in the UK | Levels of physical activity, mental health and mental wellbeing | The 21-item Becks Anxiety Inventory (BAI), BDI, The short Warwick-Edinburgh Mental Well-being Scale | To investigate the cross-sectional association between physical activity levels with depressive symptoms, anxiety symptoms, and positive mental well-being in a sample of the UK public social distancing owing to COVID-19 |
| Neill *et al.* 2021 | United Kingdom | 1989 | Baseline data collection began on 23rd March 2020 (commencement of the UK’s lockdown period) and closed on 24th April 2020 | Anxiety and depressive symptoms | GAD-7, PHQ-9 | To assess and monitor the psychosocial impact of the COVID-19 pandemic on UK residents |
| Pieh *et al.* 2021 | United Kingdom | 1006 | Four weeks after the imposition of lockdown (quarantine) measures (24 April) | Mental health related quality of life, well-being, depression anxiety, perceived stress and insomnia | WHO-Quality of Life, WHO-5: Well-Being Index, PHQ-9, GAD-7, PSS-10, ISI | To evaluate a broad set of mental health and well-being indicators |
| Rettie & Daniels, 2021 | United Kingdom | 842 | The recruitment window was open from April 17, 2020, to April 26, nearly three weeks after the stay at home order. (10-day period in the early “lockdown” phase of the pandemic) | Intolerance of uncertainty, depression, anxiety and coping strategies | Intolerance of Uncertainty Scale, PHQ-8 and GAD-7, and Brief Coping Orientation to problems experienced | To investigate whether mental health difficulties during the COVID-19 can be predicted by intolerance of uncertainty, and determine whether coping responses mediate this relationship |
| Budimir *et al.* 2021 | United Kingdom and Austria | 2011, 1005 for Austria and 1006 for the UK | After 4 weeks of quarantine in Austria as well as in the UK. (survey started on 10 April 2020 in Austria and on 21 April 2020 in the UK). Both surveys lasted 10 days | Depressive, anxiety and insomnia symptoms | PHQ-9, GAD-7, ISI, Quality of Life (WHOQOL-BREF), WHO well-being questionnaire (WHO-5), PSS-10 | To measure severe mental health symptoms, and compare them in two countries affected by COVID-19 to different degrees of severity. In addition to the severe measures of depression, anxiety and insomnia, they also measured the perceived stress, well-being and quality of life of individuals with severe depression, anxiety and insomnia |

*The Patient Health Questionnaire (PHQ), The Generalized Anxiety Disorder Scale (GAD), the Depression, Anxiety and Stress Scale (DASS), and the Hospital Anxiety and Depression Scale (HADS), General Health Questionnaire (GHQ), Perceived Stress Scale (PSS), the COVID-19 Peritraumatic Distress Index (CPDI), the Insomnia Severity Index (ISI), the Impact of Event Scale (IES), the Beck Depression Inventory (BDI)

**Table 2: A brief summary of the cohort studies included in this review.**

|  | Country | Participants (N.) | Quarantine Period | Measures | Assessment tool | Main objectives |
| --- | --- | --- | --- | --- | --- | --- |
| Salfi *et al.* 2020 | Italy | 2701 | Participants were first-time recruited during the third week of the lockdown period. Subsequently, a follow-up evaluation was carried out during the seventh week of restraining measures | Sleep quality and disturbances, severity of insomnia symptoms, symptoms of depression, perceived stress | The Pittsburgh Sleep Quality Index, ISI, BDI, PSS-10, and the State-Anxiety Inventory. | To show the trajectories of the progress of sleep and psychological well-being within the same population during the extended lockdown period. To address the different progression of sleep disturbances and psychological symptoms for men and women |
| Velotti *et al.* 2021 | Italy | 1,330 participants at Time 1 (at the beginning of the lockdown) and 308 participants at Time 2 (a few days before the end of the lockdown) | An online survey was created and diffused online 3 days after the beginning of the confinement.  Three days before the end of the national lockdown | The perceived level of loneliness, emotion dysregulation, depression and anxiety | University of California, Los Angeles Loneliness scale, Difficulties in Emotion Regulation Scale−18 items, and DASS-21 | To test the hypotheses that an increased level of loneliness experienced during COVID-19 confinement was predictive of internalising symptoms and that this pathway was mediated by emotion dysregulation levels |
| Ozamiz-Extebarria *et al.* 2020 | Spain | Two periods of the health crisis,  1,112 of the participants completed the questionnaire between the 11th and 18th of March and 881 between the 2nd and 12th of April | Two stages: 1) the time at which the government declared the state of emergency; 2) 2-3 weeks later, when people had been in lockdown for 20 days | Depression, anxiety and stress | DASS-21 | To measure the levels of stress, anxiety and depression in a sample of people from the Basque Autonomous Community in Northern Spain, a region highly affected by COVID-19 |
| Ausin *et al.* 2021 | Spain | 1014 | Two different moments in time: after two and five weeks after the declaration of the emergency state and stay at home order (21-29 March and 13-27 April) | Loneliness, depressive symptoms, anxiety and post-traumatic symptoms | UCLA Loneliness Scale, PHQ-2, GAD-2 and Civilian version of the Post-traumatic Stress Disorder Checklist | To examine the role of gender in the psychological impact of the Covid-19 pandemic |
| O’Connor *et al.* 2021 | United Kingdom | 3077 | Findings for waves 1 (31 March to 9 April 2020), 2 (10 April to 27 April 2020) and 3 (28 April to 11 May 2020) | Pre-existing mental health problems, suicide attempts and self-harm, suicidal ideation, depression, anxiety, defeat, entrapment, mental well-being and  loneliness | PHQ, GAD-7, 7-item Short Warwick Edinburgh Mental Well-Being Scale (SWEMWBS), the UCLA 3-item scale | To investigate the trajectory of mental health and well-being during the first 6 weeks of lockdown in adults in the UK |
| Zavlis *et al.* 2021 | United Kingdom | 2025 participants in wave 1 and 1406 in wave 2 | Wave one from the 23 March–28 March 2020 and wave 2 from the 22 April–1 May 2020) | Depression generalised anxiety, trauma symptoms and measures of COVID-specific anxiety | PHQ-9; GAD-7 and International Trauma Questionnaire | To understand how specific facets of the current pandemic are associated with psychopathological symptoms |
| Cheval *et al.* 2021 | France and Switzerland | 267 (wave 1) and 110 participants (wave 2) | The first questionnaire was launched on Monday, 30 March, 2 weeks after the start of the lockdown measures in France and Switzerland. The second questionnaire was launched two weeks later on Monday, 13 April (i.e., fourth week of lockdown). Both questionnaires remained open for 8 days | Physical activity, physical and mental health, anxiety, and depressive symptoms | The International Physical Activity Questionnaire, different adapted items of the Patient-Reported Outcomes Measurement Information System (PROMIS) | To assess, relative to before the COVID-19 lockdown, changes in physical activity and sedentary behaviour during commuting and leisure time during the COVID-19 lockdown, and to investigate whether changes in physical activity and sedentary behaviour during a 2-week period of the lockdown were associated with changes in health indicators |

*The Patient Health Questionnaire (PHQ), The Generalized Anxiety Disorder Scale (GAD), the Depression, Anxiety and Stress Scale (DASS), and the Hospital Anxiety and Depression Scale (HADS), General Health Questionnaire (GHQ), Perceived Stress Scale (PSS), the COVID-19 Peritraumatic Distress Index (CPDI), the Insomnia Severity Index (ISI), the Impact of Event Scale (IES), the Beck Depression Inventory (BDI)

**Table 3. Weights of the connections**
